# Fatty acid synthesis promotes mtDNA release via ETS1-mediated oligomerization of VDAC1 facilitating endothelial dysfunction in sepsis-induced lung injury

**DOI:** 10.1038/s41418-025-01524-5

**Published:** 2025-05-14

**Authors:** Shiyuan He, Tingting Pan, Rui Tian, Qian He, Decui Cheng, Hongping Qu, Ranran Li, Ruoming Tan

**Affiliations:** Department of Critical Care Medicine, Ruijin Hospital, Shanghai Jiao Tong University School of Medicine, Shanghai, 200025 PR China

**Keywords:** Proteolysis, Proteomics

## Abstract

Sepsis involves endothelial cell dysfunction leading to the development of lung injury. Fatty acid synthesis contributes to the development of inflammatory injury in sepsis. However, the regulatory mechanisms of fatty acid synthesis-related endothelial activation remain unclear. In this study, we found that fatty acid synthesis in patients with sepsis was greatly disordered. Inhibition of fatty acid synthesis significantly alleviated sepsis-induced endothelial damage and lung injury both in vitro and in vivo. We further found that the release of mtDNA participated in fatty acid synthesis-related regulation of endothelial inflammatory and coagulation activation. Mechanistically, fatty acid synthesis promoted the oligomerization of voltage-dependent anion channel 1 (VDAC1) via ETS proto-oncogene 1 (ETS1)-mediated inhibition of VDAC1 ubiquitination, thereby leading to the increased release of mtDNA and subsequent activation of cGAS-STING signaling and pyroptosis in endothelial cells. Our findings revealed that fatty acid synthesis promoted endothelial dysfunction through mtDNA release, providing new insight into the therapeutic strategies for treating sepsis-associated lung injury.

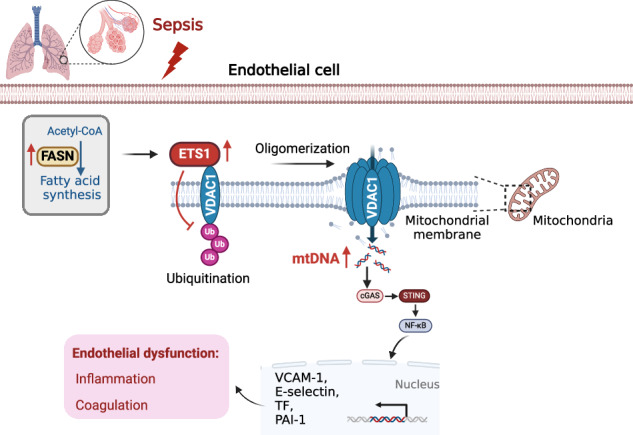

## Introduction

Sepsis is defined as a life-threatening multi-organ dysfunction resulting from a dysregulated host response to infection [1]. With an overall mortality rate of 20% to 40% [2], sepsis has become a global health crisis and is one of the major challenges of health care worldwide [3–5]. The lung is the most vulnerable target organ in sepsis and acute lung injury is the leading cause of death in septic patients [6–8]. The main pathological features of septic lung injury are the diffuse alveolar damage caused by inflammation as well as the formation of microthrombi in the pulmonary circulation induced by coagulation disorders [9, 10]. Vascular endothelial cells (ECs) are the major interface for the exchange of substances between blood and tissue and play an important role in the systemic inflammatory response during sepsis [11]. The onset of sepsis drives the pro-inflammatory activation of ECs to express adhesion molecules to facilitate leukocyte infiltration into underlying tissues, causing organ injury [12]. Additionally, ECs actively participate in the coagulation disorders, promoting the formation of thrombosis [11, 13]. Persistent and excessive activation of ECs leads to uncontrolled inflammatory and coagulation responses, which is a critical node in the occurrence and progression of sepsis-induced lung injury [14].

Lipid metabolic alterations as well as the elevation of circulating free fatty acid (FFAs) are important characteristics underlying the pathogenesis of sepsis [15]. Under septic conditions, various inflammatory factors rapidly induce the synthesis of FFAs and triglycerides, resulting in lipid accumulation [16]. The circulating levels of FFAs are directly correlated with the severity and mortality of septic patients [17]. The accumulation of excess fatty acids can lead to cellular dysfunction including the activation of inflammatory pathways, overwhelmed autophagy, mitochondrial dysfunction, and cell death [18–20]. Mitochondria is the center of cellular energy metabolism. The dysregulation of lipid homeostasis plays important roles in damaging mitochondrial integrity [21]. Once disrupted, the mitochondrial DNA (mtDNA) is released from the mitochondria to the cytosol and even the extracellular compartments. mtDNA acts as one of the major damage-associated molecular patterns (DAMPs) in triggering cellular immune responses and plays an important role in sepsis-induced multi-organ dysfunction [22–25]. Hilary et al. have demonstrated a potential link between plasma mtDNA and lung injury in septic patients [23]. However, whether the release of mtDNA plays a role in fatty acid synthesis-related endothelial dysfunction in sepsis remains elusive.

In this study, we for the first time demonstrated that overwhelmed fatty acid synthesis induced EC dysfunction in sepsis via increasing the levels of cytosolic mtDNA. We found that dysregulated fatty acid metabolism was positively correlated with the plasma levels of mtDNA in septic patients. Mechanistically, fatty acid synthesis induced the upregulation of ETS proto-oncogene 1 (ETS1), which facilitated the oligomerization of voltage-dependent anion channel 1 (VDAC1) on mitochondrial membrane by inhibiting the ubiquitination of VDAC1 monomers. VDAC1 oligomerization promoted the release of mtDNA to induce EC dysfunction via activating cGAS-STING signaling pathway, thereby leading to lung injury during sepsis. These findings pointed out the potential of fatty acid synthesis-related mtDNA release as a novel therapeutic target for treating sepsis-related EC dysfunction and lung injury.

## Results

### The dysregulation of fatty acid metabolism in septic patients

**Fig. 1:**
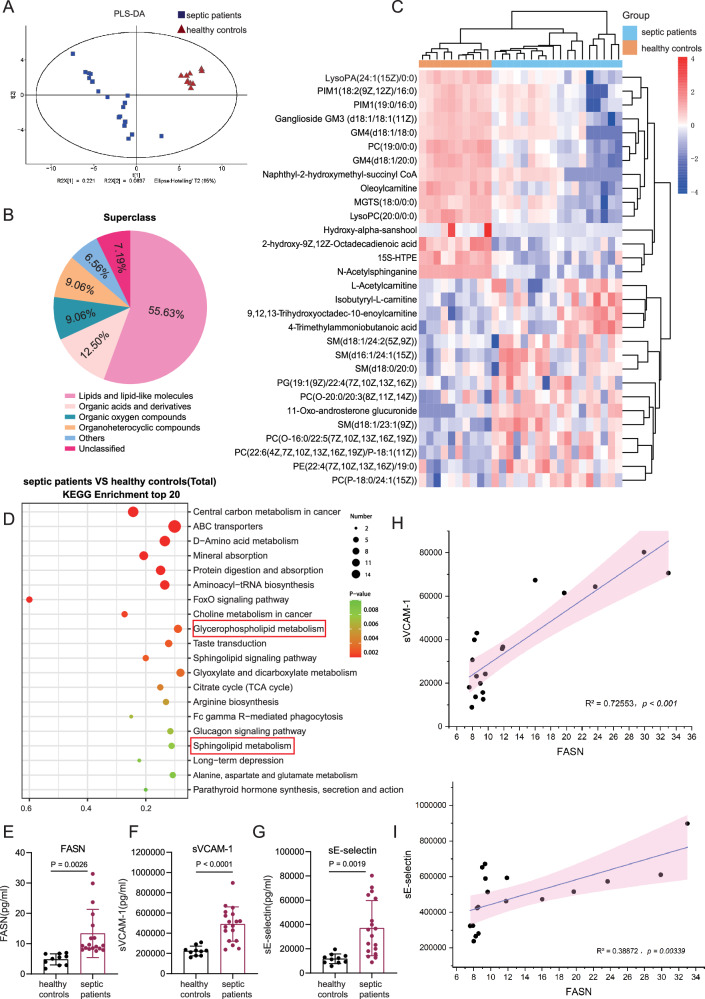
The dysregulation of fatty acid metabolism in septic patients. **A** Partial least squares discriminant analysis (PLS-DA) showing the separation of septic patients from healthy controls. **B** Pie charts of Superclass differential metabolites. **C**, **D** Heatmap and KEGG enrichment of metabolic analysis data in septic patients. **E**–**G** The levels of FASN, sVCAM-1, and sE-selectin were measured by ELISA in healthy controls (*n* = 10) and septic patients (*n* = 18). **H, I **The correlation analysis of FASN with sVCAM-1 and sE-selectin. All data were expressed as the mean ± SD. Student’s *t* test was used for (**E**–**G**). Simple linear regression and Spearman correlation coefficients were used for (**H**, **I**).

Metabolomic analysis was performed on the plasma samples of patients with sepsis and healthy controls. The characteristics of septic patients and healthy controls were shown in Supplementary Table 1. Septic patients with different severity were included. The metabolomic analysis results showed that compared to healthy controls, metabolic intermediates in septic patients were greatly altered, which included lipids and lipid-like metabolites (Fig. 1A, B). And glycerophospholipid and sphingomyelins were showed significant changes in lipids and lipid-like differential metabolites (Fig. 1C). It has been reported that some of glycerophospholipid and sphingomyelins were considered as therapy target or prognostic biomarkers for sepsis [26–28]. Additionally, Kyoto-Encyclopedia of Genes and Genomes (KEGG) analysis results also showed that the differently regulated metabolites were enriched in the signaling pathways related to glycerophospholipid metabolism and sphingolipid metabolism (Fig. 1D). In cells, fatty acid synthesis-derived fatty acids and their derivatives are the substrates for the synthesis of glycerophospholipid and sphingomyelins, which plays an important role in many diseases [29, 30]. To examine the status of fatty acid synthesis, the plasma levels of fatty acid synthase (FASN), the key enzyme for fatty acid synthesis, in septic patients were analyzed. The results showed that compared to healthy controls, the levels of FASN in plasma were significantly increased in patients with sepsis (Fig. 1E). Additionally, the increased levels of soluble E-selectin (sE-selectin) and soluble vascular cell adhesion molecule-1 (sVCAM-1) indicated the activation of endothelial cells in septic patients (Fig. 1F, G). The correlation analysis results showed that FASN was positively correlated with endothelial activation-related markers sVCAM-1 and sE-selectin (Fig. 1H, I). These data revealed that the overwhelmed fatty acid synthesis in septic patients may contribute to the pathogenesis of sepsis-induced EC dysfunction.

### Inhibition of fatty acid synthesis ameliorated endotoxemia-induced lung injury and EC dysfunction in vivo and vitro

**Fig. 2:**
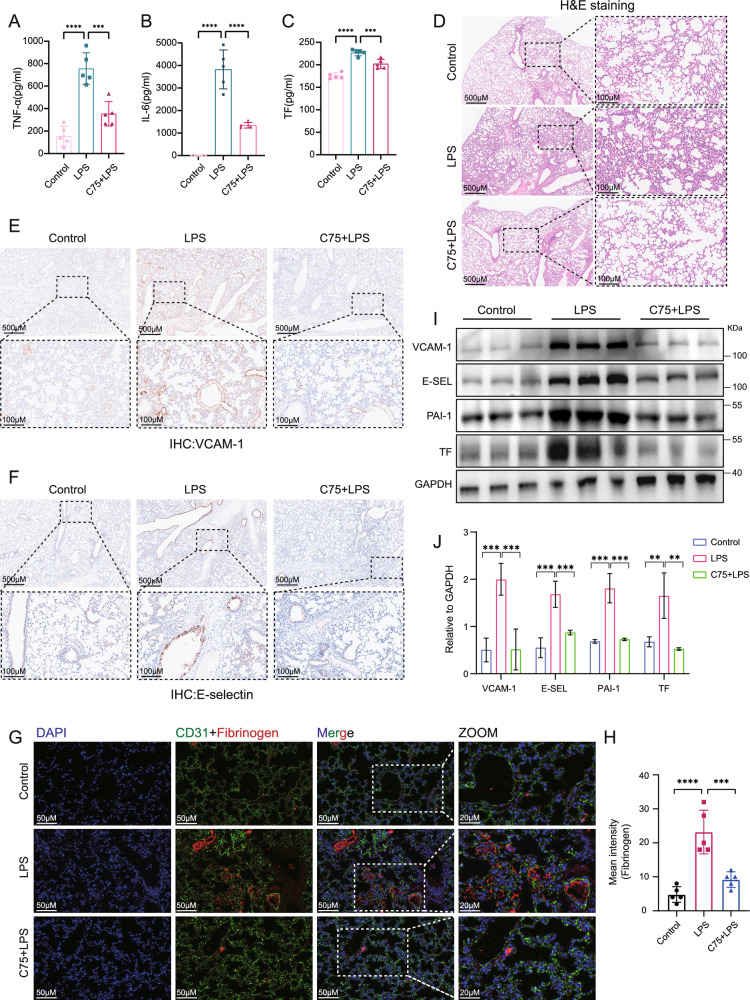
Inhibition of fatty acid synthesis ameliorated endotoxemia-induced lung injury and EC activation in vivo. The C57BL/6N mice were i.p. injected with LPS (10 mg/kg) with or without C75 (10 mg/kg, i.p.) pretreatment for 1 h (*n* = 5). **A–C** The levels of TNF-α, IL-6, and TF in mice plasma were measured by ELISA (*n* = 5). **D** H&E staining showing the tissue injury in the lung (*n* = 5). **E**, **F** IHC staining showing the levels and distribution of VCAM-1 and E-selectin in the lung tissues (*n* = 5). **G**, **H** Immunofluorescent staining showing the levels of fibrinogen (anti-fibrinogen, Red) in lungs from septic mice pretreated with or without C75 (*n* = 5). CD31 (anti-CD31, Green) was used to label endothelial cells and nuclei were stained in blue with DAPI. **I**, **J** Immunoblot showing the expression levels of VCAM-1, E-selectin, PAI-1, and TF in the lung tissues in mice (*n* = 3). All data were expressed as the mean ± SD. Comparison among three or more groups was analyzed by one-way ANOVA. ns no significance, *p < 0.05, **p < 0.01, ***p < 0.001, and ****p < 0.0001.

To investigate the effects of fatty acid synthesis on sepsis-induced lung injury, mice were intraperitoneally injected with FASN inhibitor C75 prior to lipopolysaccharide (LPS) challenge. The plasma levels of inflammatory cytokines TNF-α and IL-6 as well as coagulation-related marker tissue factor (TF) in septic mice were significantly reduced upon FASN inhibition (Fig. 2A–C). H&E staining showed that lung tissue injury in septic mice was mitigated upon C75 administration (Fig. 2D). Additionally, the vascular pro-inflammatory responses were inhibited by C75 treatment as indicated by the reduced intensity of VCAM-1 and E-selectin in septic lung tissues (Fig. 2E, F). Moreover, the immunofluorescent staining showed that the levels of fibrinogen (coagulation factor I) which is a major player in thrombus formation during coagulation were decreased in septic lung tissues after C75 treatment (Fig. 2G, H). Western blot results confirmed that LPS-induced upregulations of vascular adhesion molecules VCAM-1 and E-selectin as well as coagulation-related molecules plasminogen activator inhibitor-1 (PAI-1) and TF were significantly inhibited upon C75 pretreatment (Fig. 2I, J). These results indicated that fatty acid synthesis plays an important role in sepsis-induced lung injury via promoting EC activation and coagulation disorder in mice.

**Fig. 3:**
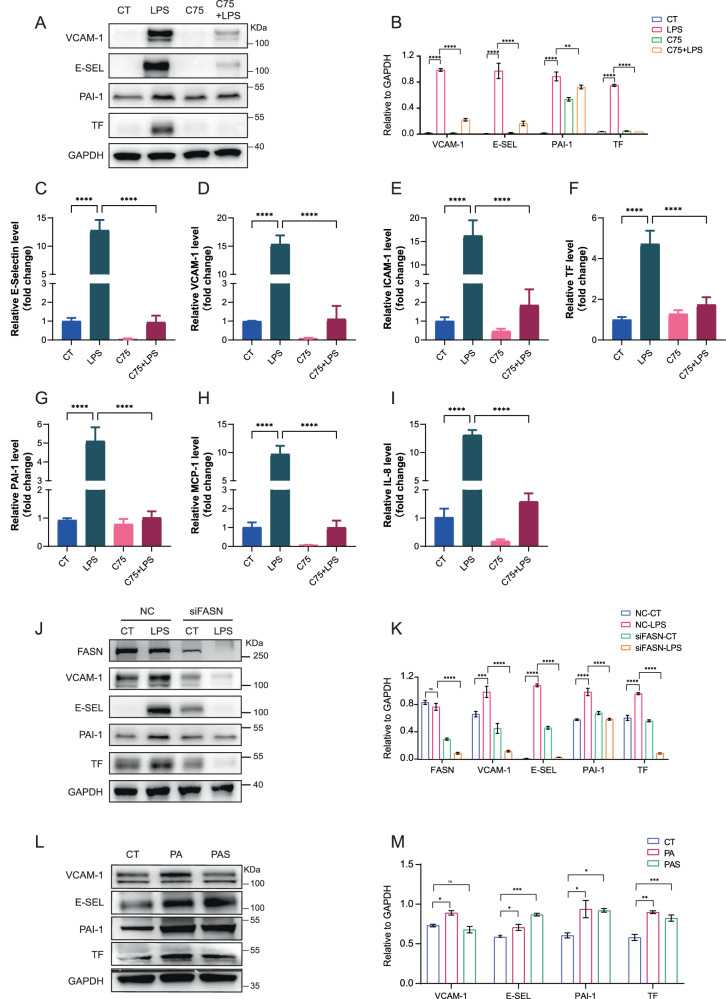
Inhibition of fatty acid synthesis ameliorated LPS-induced EC dysfunction in vitro. **A**, **B** HUVEC were pretreated with FASN inhibitor C75 for 30 min before LPS stimuli for 4 h. Protein levels of VCAM-1, E-selectin, PAI-1 and TF were analyzed by western blot (*n* = 3). **C**–**I** The mRNA levels of E-selectin, VCAM-1, ICAM-1, TF, PAI-1, MCP-1 and IL-8 with C75 treatment were detected by RT-qPCR (*n* = 3). **J**, **K** HUVEC were transfected with siRNA specific to FASN before LPS stimuli for 4 h. Protein levels of FASN, VCAM-1, E-selectin, PAI-1 and TF were analyzed by western blot (*n* = 3). **L**, **M** Immunoblot showing the expression levels of VCAM-1, E-selectin, PAI-1, and TF with PA (10 µM) or PAS (20 µM) (*n* = 3). All data were expressed as the mean ± SD. Comparison among three or more groups was analyzed by one-way ANOVA. ns no significance, *p < 0.05, **p < 0.01, ***p < 0.001, and ****p < 0.0001.

To verify the role of fatty acid synthesis in EC dysfunction, human umbilical vein endothelial cells (HUVEC) were pretreated with C75 before LPS stimulus. The results showed that LPS-induced upregulations of adhesion molecules VCAM-1 and E-selectin as well as coagulation-related molecules PAI-1 and TF were significantly inhibited upon C75 pretreatment (Fig. 3A, B). Additionally, the mRNA levels of E-selectin, VCAM-1, ICAM-1, TF, PAI-1, MCP-1, and IL-8 induced by LPS were also significantly downregulated by C75 (Fig. 3C–I). HUVEC demonstrated tolerance to LPS stimulation in this study, and notably, a 2-hour LPS exposure induced a significant increase in the protein levels of FASN (Supplementary Fig. 1A–C). The deletion of FASN also showed similar effects on LPS-induced EC activation (Fig. 3J, K). Moreover, exogenous addition of fatty acid synthesis products palmitic acid (PA) and palmitate (PAS) increased the protein levels of VCAM-1, E-selectin, PAI-1, and TF in HUVEC (Fig. 3L, M). These data indicated that overwhelmed intracellular fatty acid accumulation leads to EC activation in sepsis.

### Cytosolic mtDNA release mediated fatty acid synthesis-related EC activation in sepsis

**Fig. 4:**
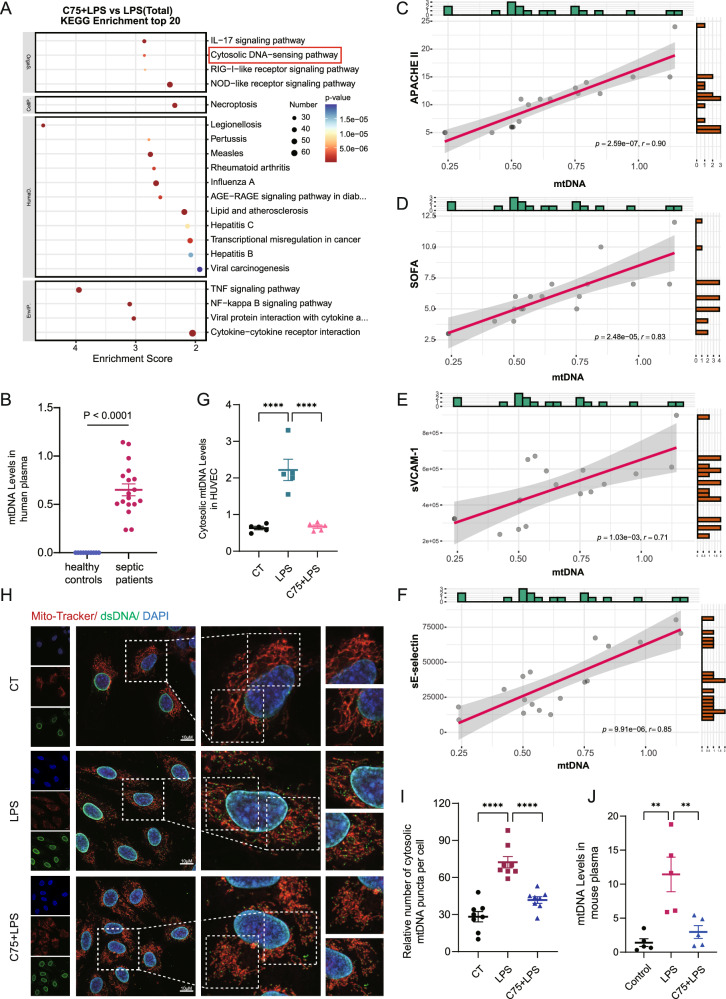
Cytosolic mtDNA release mediated fatty acid synthesis-related EC activation in sepsis. **A** KEGG analysis of genes regulated by both LPS stimulation and C75 treatment. **B** The levels of mtDNA in the plasma of septic patients (*n* = 18) were measured by RT-qPCR. **C**–**F** The correlation analysis of mtDNA with APACHE II score, SOFA score, sVCAM-1, and sE-selectin. **G** HUVEC were pretreated with C75 for 30 min before LPS stimuli for 4 h. Cytosolic mtDNA/nDNA was detected by RT-qPCR (*n* = 5). **H**, **I** HUVEC were pretreated with C75 before LPS stimuli. Confocal laser microscope assay was used to assess the levels of mtDNA (anti-dsDNA, green). Mitochondria were stained in red with Mito-Tracker and nuclei were stained in blue with DAPI. Quantitative results from 8 cells for each condition were reported. **J** The levels of mtDNA in the plasma of septic mice (*n* = 5) were measured by RT-qPCR. All data were expressed as the mean ± SD. Simple linear regression and Spearman correlation coefficients were used for (**C**–**F**). Unpaired t-test was used for the comparison between two groups. Comparison among three or more groups was analyzed by one-way ANOVA. ns no significance, *p < 0.05, **p < 0.01, ***p < 0.001, and ****p < 0.0001.

To investigate the detailed mechanisms of fatty acid synthesis-induced EC dysfunction in sepsis, transcriptome analysis was performed on HUVEC challenged by LPS with or without C75 pretreatment. The KEGG analysis results showed that the different expressed genes (DEGs) regulated by FASN inhibition in HUVEC were significantly enriched in cytosolic DNA-sensing pathway (Fig. 4A). Cytosolic mtDNA has been reported to be a major trigger of inflammatory pathways [31, 32]. We measured the levels of mtDNA in the plasma of septic patients by RT-qPCR. The results showed that plasma levels of mtDNA were significantly increased in patients with sepsis compared to those in healthy controls (*P* < 0.0001) (Fig. 4B). The correlation analysis results showed that the plasma levels of mtDNA were positively associated with APACHE II score and SOFA score as well as the levels of EC activation markers sVCAM-1 and sE-selectin (*p* < 0.0001, *p* < 0.0001, *p* = 0.00103, *p* < 0.0001, respectively) (Fig. 4C–F). These data implied the potentially detrimental effects of mtDNA on the pathogenesis of sepsis-induced EC dysfunction.

We further analyzed the effect of FASN inhibition on mtDNA release in HUVEC. The results showed that cytosolic levels of mtDNA in HUVEC were upregulated upon LPS stimuli and downregulated by C75 pretreatment (Fig. 4G). Confocal images also showed that LPS-induced increase in the levels of cytosolic mtDNA not co-localized with Mito-Tracker or DAPI was decreased upon C75 pretreatment (Fig. 4H, I). Moreover, the elevated plasma mtDNA levels in septic mice was also significantly reduced upon C75 administration (Fig. 4J). These data suggested the potential role of cytosolic mtDNA in fatty acid synthesis-induced EC activation in sepsis.

### Fatty acid synthesis promoted mtDNA-related activation of cGAS-STING signaling and pyroptosis in ECs in response to LPS

**Fig. 5:**
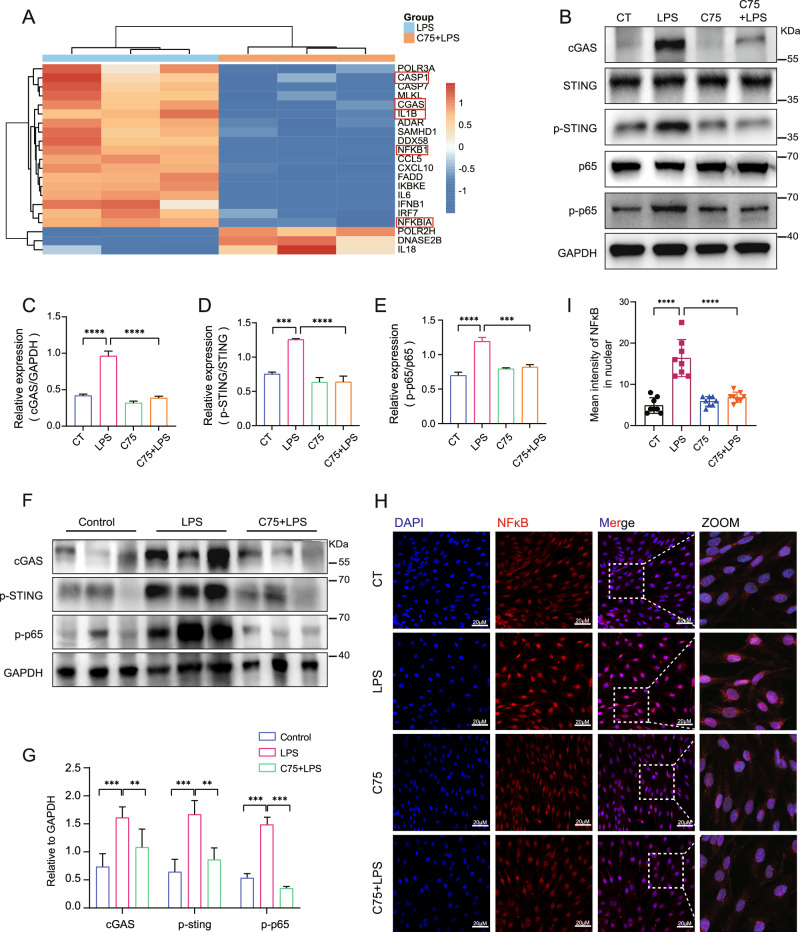
Fatty acid synthesis promoted mtDNA-related activation of cGAS-STING signaling and pyroptosis in ECs in response to LPS. **A** Heatmap of DEGs enriched in cytosolic DNA-sensing pathway. **B**–**E** HUVEC were pretreated with C75 before LPS stimuli. Protein levels of cGAS, p-STING, and p-p65 were determined by western blot (*n* = 3). **F**, **G** Representative immunoblots of cGAS, p-STING, and p-p65 in the lung tissues of septic mice pretreated with or without C75 (*n* = 3). **H**, **I** Confocal images of p65 nuclear translocation in LPS-challenged HUVEC with or without C75 pretreatment. Quantitative results from 8 cells per group are reported. All data were expressed as the mean ± SD. Comparison among three or more groups was analyzed by one-way ANOVA. ns no significance, *p < 0.05, **p < 0.01, ***p < 0.001, and ****p < 0.0001.

It has been reported that cytosolic mtDNA binds DNA pattern recognition receptors (PRRs), triggering the activation of innate immune signaling pathways [33]. The heatmap of DEGs enriched in the cytosolic DNA-sensing pathway showed that LPS-induced expression of cGAS, caspase 1, and IL-1β were significantly inhibited by FASN inhibition, suggesting the involvement of cGAS-STING signaling and pyroptosis in fatty acid synthesis-related EC activation (Fig. 5A). At protein levels, the upregulations of cGAS and phosphorylated STING by LPS were inhibited upon C75 pretreatment (Fig. 5B–D). C75 also reduced the protein levels of cleaved caspase 1 and the N-terminal of Gasdermin D (Supplementary Fig. 1D, E). In septic mice, the upregulations of cGAS and phosphorylated STING as well as cleaved caspase 1 and N-terminal of Gasdermin D in the lung tissue were all effectively decreased upon C75 administration (Fig. 5F, G; Supplementary Fig. 1F, G). Moreover, as the downstream transcription factor of cGAS-STING signaling, the phosphorylation of NF-κB subunit p65 was inhibited upon FASN inhibition (Fig. 5B, E). The confocal laser microscopy results showed that LPS-induced nuclear translocation of p65 was inhibited upon C75 pretreatment (Fig. 5H, I). These data revealed that fatty acid synthesis promoted EC dysfunction via mtDNA-related activation of cGAS-STING signaling and pyroptosis.

### VDAC1 was associated with fatty acid synthesis-related mtDNA release and EC dysfunction during sepsis

**Fig. 6:**
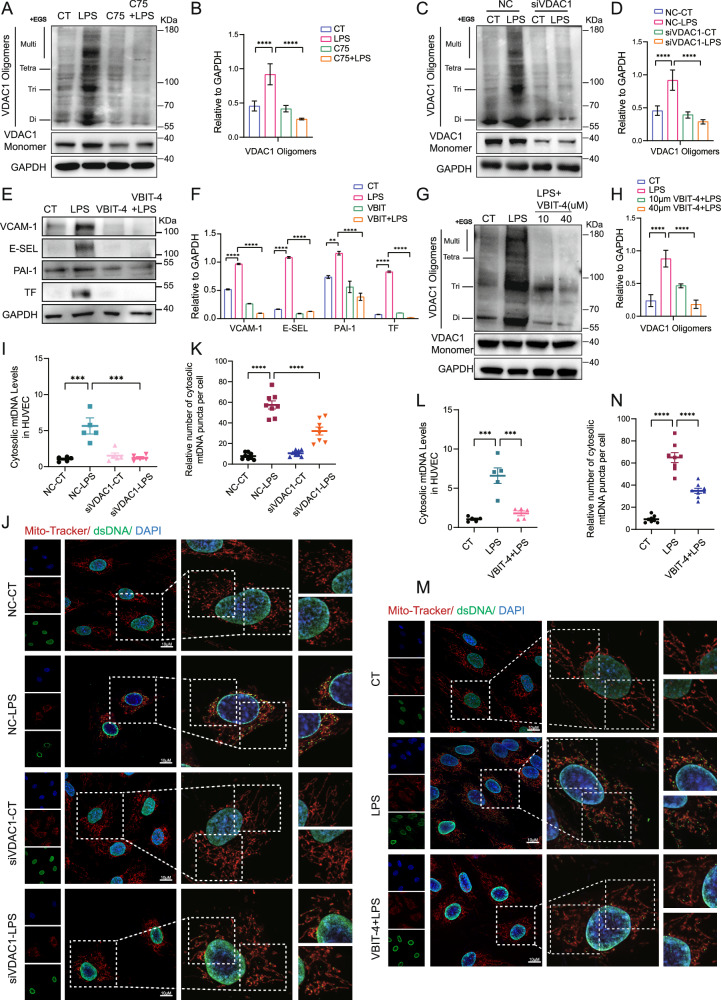
VDAC1 was associated with fatty acid synthesis-related mtDNA release and EC dysfunction during sepsis. **A**, **B** HUVEC were pretreated with C75 before LPS stimuli. The levels of VDAC1 oligomerization were detected by western blot (*n* = 3). **C**, **D** VDAC1 were knocked down with siRNA before LPS stimuli. The levels of VDAC1 oligomerization were detected by western blot (*n* = 3). **E**, **F** Immunoblot showing the expression levels of VCAM-1, E-selectin, PAI-1 and TF in HUVEC pretreated with VBIT-4 (40 µM) before LPS stimuli (*n* = 3). **G**, **H** HUVEC were stimulated with LPS in the presence or absence of VBIT-4 (10 µM, 40 µM). The protein levels of VDAC1 oligomerization were analyzed by western blot (*n* = 3). **I** VDAC1 in HUVEC was knocked down with siRNA before LPS stimuli for 4 h. Cytosolic mtDNA/nDNA was detected by RT-qPCR (*n* = 5). **J**, **K** VDAC1 was knocked down before LPS stimuli. Confocal laser microscope assay was used to assess the levels of mtDNA (anti-dsDNA, green). Mitochondria were stained in red with Mito-Tracker and nuclei were stained in blue with DAPI. Quantitative results from 8 cells for each condition were reported. **L** HUVEC were stimulated with LPS in the presence or absence of VBIT-4 (40 µM). Cytosolic mtDNA/nDNA was detected by RT-qPCR (*n* = 5). **M**, **N** HUVEC were pretreated with VBIT-4 (40 µM) before LPS stimuli. Confocal laser microscope assay was used to assess the levels of mtDNA (anti-dsDNA, green). Mitochondria were stained in red with Mito-Tracker and nuclei were stained in blue with DAPI. Quantitative results from 8 cells for each condition were reported. All data were expressed as the mean ± SD. Comparison among three or more groups was analyzed by one-way ANOVA. ns no significance, *p < 0.05, **p < 0.01, ***p < 0.001, and ****p < 0.0001.

Voltage-dependent anion channel 1 (VDAC1) has been considered as an important role in the release of mtDNA via its oligomerization [34]. We found that the inhibition of FASN reduced the level of homo-oligomerization of VDAC1 (Fig. 6A, B). We further examined whether VDAC1 oligomerization plays a role in EC dysfunction, The result showed that the deletion of VDAC1 or pretreatment of VBIT-4 (the inhibitor of VDAC1 oligomerization) in HUVEC before LPS challenge effectively inhibited VDAC1 oligomerization as well as the upregulations of VCAM-1, E-selectin, PAI-1, and TF (Fig. 6C–H; Supplementary Fig. 2A, B). The mRNA levels of IL-8, MCP-1, ICAM-1, VCAM-1, E-selectin, PAI-1, and TF in HUVEC stimulated by LPS were also downregulated by VBIT-4 pretreatment (Supplementary Fig. 2C–I). Moreover, the cytosolic mtDNA levels in HUVEC induced by LPS stimuli were significantly reduced upon the knockdown of VDAC1 and VBIT-4 pretreatment respectively (Fig. 6I–N). These data indicated that fatty acid synthesis-related promotion of the release of mtDNA and EC dysfunction in sepsis may be associated with VDAC1 oligomerization.

### ETS1 participated in fatty acid synthesis-related mtDNA release and EC damage induced by LPS

**Fig. 7:**
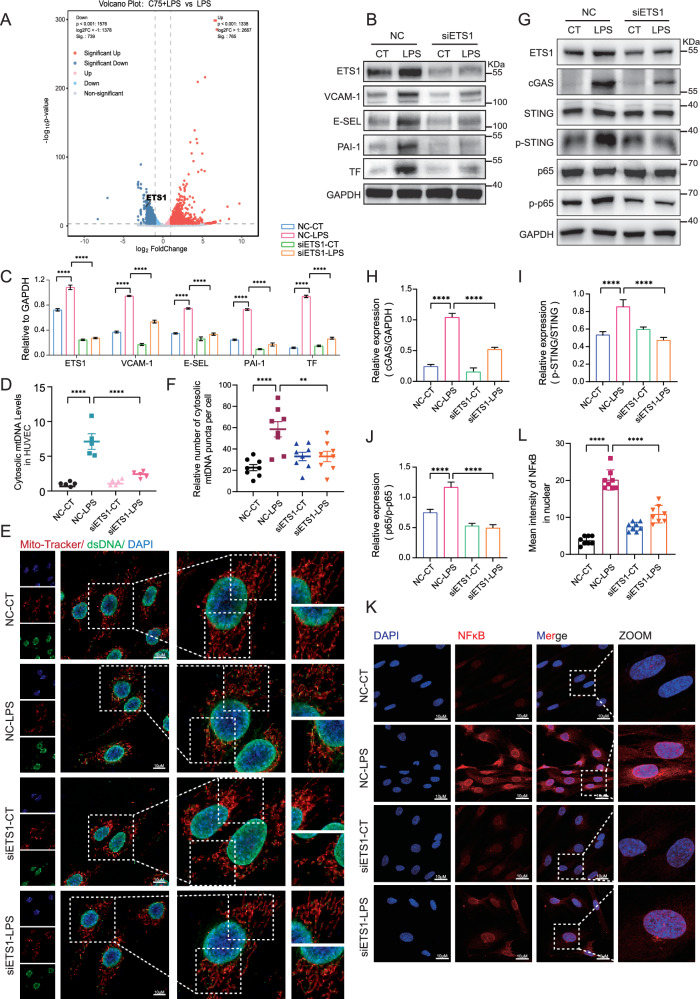
ETS1 participated in fatty acid synthesis-related mtDNA release and EC damage induced by LPS. **A** Volcano plot of differently expressed genes. **B**, **C** HUVEC were transfected with siETS1 before LPS stimuli for 4 h. Expression levels of ETS1, VCAM-1, E-selectin, PAI-1, and TF were analyzed by western blot (*n* = 3). **D** ETS1 in HUVEC was knocked down with siRNA before LPS stimuli for 4 h. Cytosolic mtDNA/nDNA was detected by RT-qPCR (*n* = 5). **E**, **F** ETS1 in HUVEC was knocked down with siRNA before LPS stimuli. Confocal laser microscope assay was used to assess the levels of mtDNA (anti-dsDNA, green). Mitochondria were stained in red with Mito-Tracker and nuclei were stained in blue with DAPI. Quantitative results from 8 cells for each condition were reported. **G**–**J** HUVEC were pretreated with siRNA specific to ETS1 before LPS stimuli. Protein levels of cGAS, p-STING, and p-p65 were determined by western blot (*n* = 3). **K**, **L** Confocal images of p65 nuclear translocation in LPS-challenged HUVEC with or without ETS1 knockdown. Quantitative results from 8 cells per group are reported. All data were expressed as the mean ± SD. Comparison among three or more groups was analyzed by one-way ANOVA. ns, no significance, *p < 0.05, **p < 0.01, ***p < 0.001, and ****p < 0.0001.

ETS proto-oncogene 1 (ETS1) is a transcription factor that play important roles in cell proliferation, differentiation, and cellular functions in several diseases [35]. However, its role in metabolism-related regulation of EC functions remains unclear. The transcriptome analysis results showed that the expression of ETS1 was significantly downregulated upon the treatment of C75 (Fig. 7A), which was also confirmed at protein levels (Supplementary Fig. 3A, B). The deletion of ETS1 in HUVEC effectively inhibited the upregulations of VCAM-1, E-selectin, PAI-1, and TF induced by LPS in both protein and mRNA levels (Fig. 7B, C; Supplementary Fig. 3C–J), suggesting the regulatory importance of ETS1 in EC activation during sepsis. Moreover, the cytosolic mtDNA levels in HUVEC induced by LPS stimuli were reduced upon the knockdown of ETS1 (Fig. 7D–F). The deletion of ETS1 decreased the levels of cGAS, phosphorylated STING and the phosphorylation of NF-κB subunit p65 as well as cleaved caspase 1 and the N-terminal of Gasdermin D induced by LPS (Fig. 7G–J; Supplementary Fig. 3K, L). Additionally, the confocal laser microscopy results also showed that LPS-induced nuclear translocation of p65 was inhibited upon ETS1 deficiency (Fig. 7K, L). These data indicated that fatty acid synthesis-related promotion of the release of mtDNA and EC dysfunction during sepsis may be mediated by ETS1.

### ETS1 promoted VDAC1 oligomerization to facilitate mtDNA release and EC activation via limiting VDAC1 ubiquitination

**Fig. 8:**
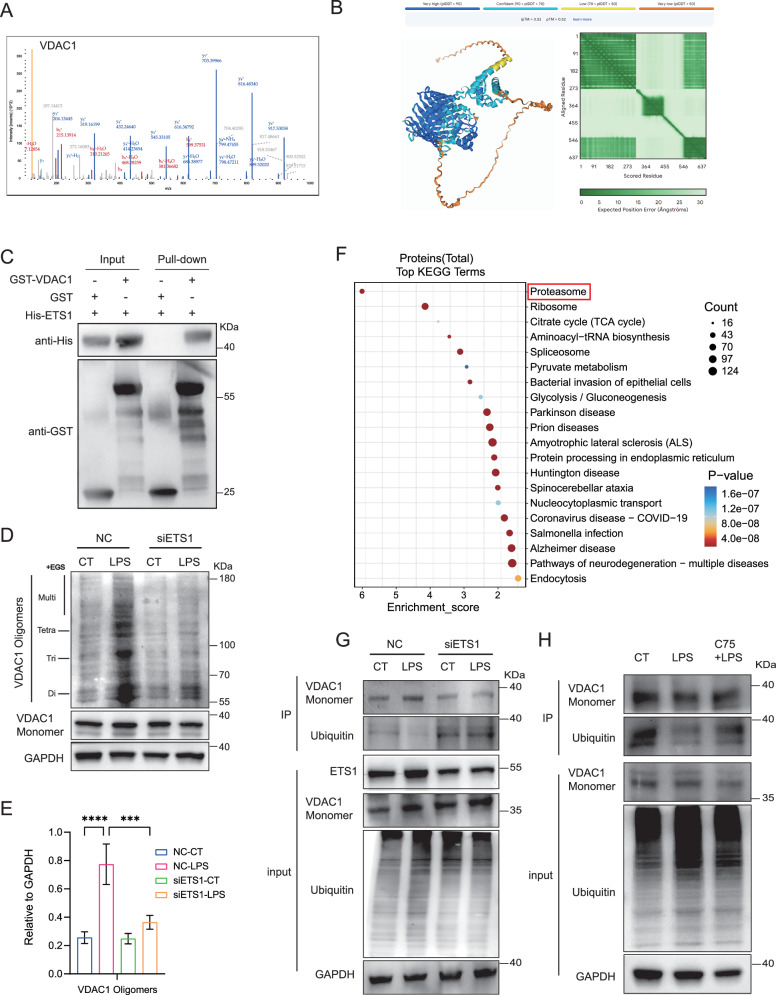
ETS1 promoted VDAC1 oligomerization to facilitate mtDNA release and EC activation via limiting VDAC1 ubiquitination. **A** CoIP-MS image of VDAC1. **B** Molecular docking showing the interaction between ETS1 and VDAC1. **C** Immunoblot showing the binding of ETS1 with VDAC1 by GST pull-down (*n* = 3). **D**, **E** HUVEC were transfected with siRNA specific to ETS1 before LPS stimuli. The levels of VDAC1 oligomerization were detected by western blot (*n* = 3). **F** KEGG analysis of proteins binding to ETS1. **G**, **H** HUVEC were transfected with siRNA specific to ETS1 or pretreated with C75 before LPS stimuli. The ubiquitination levels of VDAC1 were detected by western blot (*n* = 3). All data were expressed as the mean ± SD. Comparison among three or more groups was analyzed by one-way ANOVA. ns no significance, *p < 0.05, **p < 0.01, ***p < 0.001, and ****p < 0.0001.

Proteins that interacted with ETS1 in HUVEC were screened to explore the mechanisms of ETS1 in regulating mtDNA release in ECs during sepsis. We found that VDAC1 was identified to interact with ETS1 (Fig. 8A). Subsequently, the direct interaction between ETS1 and VDAC1 was verified by molecular docking and GST pull-down analysis. The results of molecular docking analysis using AlphaFold3 showed that ipTM + pTM > 0.75, suggesting the strong binding between ETS1 and VDAC1 (Fig. 8B). The GST pull-down results further confirmed the direct binding of ETS1 to VDAC1 protein (Fig. 8C). The immunoprecipitation results showed increased interaction between ETS1 and VDAC1 in HUVEC upon LPS challenge, which was significantly reduced by FASN inhibition (supplementary Fig. 4A). Additionally, the oligomerization level of VDAC1 was increased by LPS stimuli, which was inhibited upon ETS1 deficiency (Fig. 8D, E). It has been reported that the oligomerization of VDAC1 and subsequent mtDNA release are restricted by the ubiquitination of VDAC1 in liver fibrosis [36]. The KEGG analysis showed that proteins that interacted with ETS1 in HUVEC were enriched in proteasome-related pathway, indicating the potential relations of ETS1 with protein ubiquitination and proteasome-dependent degradation (Fig. 8F). To verify the regulation of VDAC1 ubiquitination by ETS1, ETS1 was knocked down in HUVEC before LPS stimuli. The results showed that the ubiquitination level of VDAC1 was increased upon ETS1 deletion (Fig. 8G). Conversely, the overexpression of ETS1 in HUVEC decreased the level of VDAC1 ubiquitination (Supplementary Fig. 4B). Additionally, the inhibition of FASN increased the ubiquitination level of VDAC1 in HUVEC, leading to the decreased level of VDAC1 oligomerization (Fig. 8H). These data indicated that fatty acid synthesis may increase VDAC1 oligomerization via ETS1-mediated reduction of VDAC1 ubiquitination, thereby promoting mtDNA release-related EC activation during sepsis.

## Discussion

**Fig. 9:**
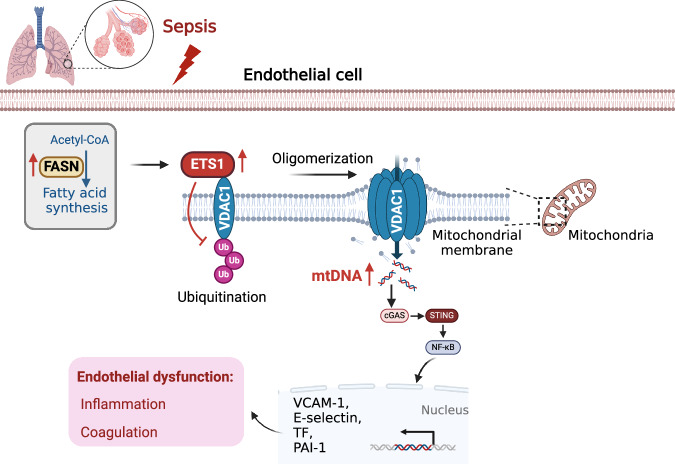
Mechanistic diagram. In sepsis, overwhelmed synthesis of fatty acids promotes ETS1 expression which limited the ubiquitination of VDAC1 and promoted its oligomerization. VDAC1 oligomerization facilitates the release mtDNA into the cytosol to activate cGAS-STING signaling, leading to endothelial dysfunction in sepsis-induced lung injury.

Sepsis is closely related to metabolic disorders. FFAs, glucose, cholesterol, and oxidized phospholipids are important metabolism-associated molecular patterns (MAMPs). MAMPs play pivotal roles in the pathogenesis of sepsis. However, the mechanism of how the metabolic process regulates EC dysfunction in sepsis remains unclear. In this study, we demonstrated that fatty acid synthesis induced EC activation via promoting cytosolic mtDNA-related activation of cGAS-STING signaling and pyroptosis, thereby leading to lung injury during sepsis. Additionally, we found that the plasma levels of mtDNA in septic patients were positively correlated with the severity of sepsis as well as the levels of EC damage-related markers including sVCAM-1 and sE-selectin. Our findings revealed the fatty acid synthesis-mediated mtDNA release may be the potential therapeutic target for treating sepsis-related EC dysfunction and lung injury (Fig. 9).

Lipid metabolism has increasingly become the research focus in various diseases. Sepsis as a disease of metabolic imbalance showed the evident disorders of lipid metabolism [15, 37]. Various inflammatory factors could induce fatty acid and triglyceride synthesis in sepsis, resulting in lipid accumulation [16]. Jolien et al. also noted that the significantly increased plasma levels of FFAs were directly correlated with the severity and the mortality rate of sepsis [17]. Palmitic acid as the final metabolite of fatty acid synthesis regulates immune responses by promoting protein palmitoylation modifications [38, 39]. Additionally, palmitic acid is a key precursor of other long-chain saturated fatty acids and long-chain unsaturated fatty acids, the excessive intracellular accumulation of which can lead to lipotoxicity [40, 41]. Our previous study has shown that the inhibition of ATP citrate lyase (ACLY)-related gluco-lipogenesis could ameliorate sepsis-induced multiple organ dysfunction via inhibiting FoxO1-MYC-mediated vascular activation [42]. Intracellular fatty acids are mainly derived from de novo fatty acid synthesis [42]. FASN is the key enzyme in de novo lipogenesis (DNL) that converts acetyl-coenzyme A (CoA) and malonyl-CoA into palmitic acid [42]. It has been demonstrated that FASN-dependent intracellular fatty acid accumulation promoted the inflammatory response in neutrophils [38]. FASN inhibition attenuates inflammatory and fibrotic drivers of NASH by direct inhibition of immune and stellate cells, beyond decreasing fat accumulation in hepatocytes [43]. In the vasculature, FASN facilitated the angiogenesis of endothelial cells via the palmitoylation of mTOR [44]. However, the role of FASN in sepsis-induced vascular dysfunction remains unclear. In this study, we found that in patients with sepsis, the plasma levels of FASN were elevated and were positively correlated with the levels of EC damage-related markers sVCAM-1 and sE-selectin as well as the severity of patients, indicating the great importance of FASN in the pathogenesis of sepsis. Moreover, we confirmed that the inhibition of FASN significantly alleviated vascular activation and lung injury in septic mice. Our findings revealed the significant role of fatty acid synthesis in the pathogenesis of sepsis, providing novel therapeutic target for treating sepsis.

In addition, we found that cytosolic mtDNA plays a pivotal role in fatty acid synthesis-induced EC activation in sepsis. mtDNA is the novel DAMP that trigger the immune response in diseases. mtDNA is present in nucleoids and restricted to the mitochondrial matrix [25, 33, 45]. Upon stress, loss of mitochondrial integrity releases oxidized and cleaved mitochondrial DNA from within the mitochondria into the cytosol [45]. It has been reported that circulating mtDNA levels were significantly elevated in septic patients within 24 h of admission to the intensive care unit compared to controls [46]. In addition, Feng et al. noted that plasma mtDNA levels were elevated in septic AKI patients [47]. Serum mtDNA levels were also significantly higher in the septic shock and death groups than in the septic non-shock and non-death groups [46]. Higher plasma levels of mtDNA have been reported to be associated with the incidence of ALI and the mortality rate of septic patients [48, 49]. These data indicated that mtDNA may accurately predict the 28-day mortality rate of septic patients. We here demonstrated the positive correlations of septic patients’ mtDNA levels with the levels of EC damage-related markers sVCAM-1 and sE-selectin in plasma as well as with the severity of the patients, indicating the great importance of mtDNA in the pathogenesis of sepsis and sepsis-related vascular injury. Furthermore, we found that the inhibition of fatty acid synthesis effectively reduced the levels of cytosolic mtDNA in ECs and diminished mtDNA-related activation of cGAS-STING signaling and pyroptosis. Our findings revealed the importance of mtDNA release in regulating EC activation during sepsis, suggesting mtDNA as a new marker for the diagnosis of sepsis-related EC dysfunction.

mtDNA release is a central mechanism linking mitochondrial dysfunction and inflammation. Early understanding of mtDNA release came from studies of the mitochondrial apoptotic pathway, which requires mitochondrial outer membrane permeabilization (MOMP) for the release of cytochrome C, which initiates the apoptotic cascade [45, 50]. The Bcl-2 family of proteins (BAK and BAX) accumulated in the mitochondrial outer membrane (MOM) in response to apoptotic stimuli were the first identified MOMP mediators [45, 51–53]. Additionally, VDAC1 as the most abundant protein in MOM is considered as the mitochondria gate-keeper protein [54, 55]. mtDNA can also be released by oligomerization of VDAC1 into a pore, thereby promoting apoptosis, pyroptosis, and mitochondrial autophagy [56–58]. Different from BAK/BAX pore, VDAC oligomers can be formed in both living and apoptotic cells and require the opening of the mitochondrial permeability transition pore (mPTP) for mitochondrial inner membrane (MIM) permeabilization [34]. Recently, it has been reported that the N-terminal pore-forming Gasdermin D fragment (GSDMD-NT) rapidly damaged both inner and outer mitochondrial membranes, leading to transmembrane potential and release of mitochondrial proteins and DNA from the matrix and intermembrane space, which accelerated and enhanced pyroptosis [59]. Considering the importance of Gasdermin pore in immune and inflammatory diseases, it is worthwhile to investigate fatty acid synthesis regulation of Gasdermin pore formation in mitochondria for mtDNA release in the future.

ETS1 belongs to the family of ETS transcription factors, which plays a key regulatory role in many physiological and pathological processes by regulating cell proliferation, differentiation, apoptosis and cell-to-cell interactions [35, 60–62]. In ECs, the expression of ETS1 plays an important role in angiogenesis [63, 64]. Vascular endothelial growth factor (VEGF) regulates RNA through the transcription factor ETS1, and the arrest-release of polymerase II (RNAPII) promotes angiogenic function of ECs [63]. In our present study, we found that the inhibition of FASN reduced the upregulation of ETS1 induced by LPS. We further found that the deletion of ETS1 significantly inhibited LPS-induced EC activation, suggesting the potential involvement of ETS1 in fatty acid synthesis-related EC dysfunction in sepsis. Besides as a well-known transcription factor, ETS transcription factors are also commonly regulated by post-translational modification such as ubiquitination, sumoylation, and phosphorylation. Phosphorylation of ETS proteins is mediated by MEK/ERK signaling for promoting downstream gene expression [65]. In addition, ETS1 has been identified to be deubiquitinated by Usp9x in melanoma [66]. Except for the ubiquitination of ETS1 itself, we investigated whether ETS1 could regulate the process of protein ubiquitination. In our study, the immunoprecipitation (Co-IP) and mass spectrometry results showed that proteins binding to ETS1 were significantly enriched in proteome-related signaling and included several ubiquitination-related enzymes such as TRIM22, TRIM25, and TRIM28 (Supplementary Fig. 4C–E). This suggested that ETS1 might be involved in regulating the process of protein ubiquitination. Additionally, among the large number of mitochondrial proteins binding to ETS1, VDAC1 plays an important role in regulating mtDNA release. VDAC1 belongs to VDAC family including VDAC1, VDAC2, and VDAC3 [67, 68]. VDAC1 as the most widely expressed protein expresses on the outer mitochondrial membrane. In pathological conditions, VDAC1 overexpression leads to its oligomerization. mtDNA can be released through oligomerized VDAC1 channels, triggering a series of downstream responses [54, 69–71]. It has been reported that ubiquitination of VDAC1 restricts its oligomerization and limits mtDNA release [36, 58, 64, 72]. We here found reduced level of VDAC1 ubiquitination in ECs under septic conditions. The inhibition of fatty acid synthesis increased the ubiquitination level of VDAC1 and inhibited its oligomerization. ETS1 knockdown also upregulated the ubiquitination modification of VDAC1, thereby inhibiting its oligomerization and mtDNA release. Our findings for the first time revealed the regulatory role of ETS1 in fatty acid synthesis-related mtDNA release via modulating VDAC1 ubiquitination and oligomerization, indicating the important role of ETS1 in modulating in protein ubiquitination. The detailed mechanisms regarding the (de)ubiquitinases involved in ETS1-related regulation of ubiquitination of VDAC1 needs to be further investigated in the future study.

Collectively, we discovered for the first time that fatty acid synthesis promoted mtDNA release-related EC dysfunction via ETS1-mediated VDAC1 ubiquitination and oligomerization, thereby leading to the lung injury in sepsis. These findings provided novel diagnostic and therapeutic strategies for sepsis management.

## Materials and methods

### Patients

Patients diagnosed with sepsis were enrolled in the Department of Critical Care Medicine in Shanghai Ruijin Hospital from February 1^st^ to April 20^th^, 2023. This study protocol conformed to the ethics guidelines of the Declaration of Helsinki and was approved by the ethics committee of Ruijin Hospital. On the day of enrollment, withdrawn the peripheral blood from both septic patients and healthy volunteers. And then, collected plasma after centrifugation at 1500 rpm for 10 min and stored the samples at −80 °C for further analysis.

### Murine model of sepsis

We obtained the male C57BL/6N mice (6–8 weeks, 20–25 g) from Charles River (Beijing, China). Randomly divided the mice into experimental groups (*n* =  5 per group). No statistical methods were used for the animal sample size. Then, the mice were injected intraperitoneally with LPS (L2630, E.coli 0111:B4, Sigma Aldrich, MA, USA) (10 mg/kg body weight). In the intervention group, the mice were i.p injected with C75 (#S9819, Selleck, Shanghai, China) (10 mg/kg body weight) before LPS injection. Vehicle control mice were i.p injected with 100 µL of 0.9% saline. After 16 h of LPS challenge, anesthetized the mice and collected the blood samples. The organs were snap frozen or fixed with formalin for further examination, and then stored at −80 °C. Investigators were blinded to the group allocation for the analysis. The protocols for animal experiments were approved by the Animal Ethics Committee of Shanghai Ruijin Hospital and were in line with the International Guidelines for Care and Use of Laboratory Animals (National Academy of Sciences Health Publication No. 85-23, revised in 1996).

### Cell culture

We isolated human umbilical vein endothelial cells (HUVEC) from human umbilical cords acquired from the Department of Obstetrics with the consent of donors in Ruijin Hospital. Then, cultured in HUVEC-specific medium (1001, ScienCell, CA, USA), which was also supplemented with 5% fetal bovine serum, streptomycin and penicillin.

### Western blot assay

Proteins from HUVEC and mice tissues were lysed using RIPA buffer (#89901, Thermo fisher Scientific) supplemented with PhosSTOP and complete protease inhibitors (Merck, Beijing, China). Then, detected the proteins’ concentrations by BCA assay kit (Thermo Fisher Scientific, Waltham, MA, USA). The protein extracts were denatured at 100 °C for 5 min and separated by 10% SDS-PAGE at 110–120 V for 1.5 h. Proteins were blotted onto a PVDF membrane (162–0177, BioRad, Shanghai, China) at 200 mA for 120 min. Then, membranes were blocked with 5% bovine serum albumin (BSA) for 1 h at RT. Incubated the membranes with primary antibodies (1:1,000) overnight at 4 °C. Then incubated with specific secondary antibodies (1:5000) for 1 h at RT, and then detected by enhanced chemiluminescence (ECL). The information about antibodies was shown in Supplementary Table 2.

### Cytosolic mtDNA isolation and detection

HUVEC were lysed and centrifuged at 700 × *g* for 10 min to remove nuclei. Next, the supernatant volume normalized based on protein concentration. Cell lysate was then centrifuged at 10,000 × *g* for 30 min for cytosolic fraction isolation, which included mtDNA and nDNA [73]. In mouse and human plasma samples, DNA was extracted by using DNA Purification Kit (2918742, Thermo Fisher Scientific, Waltham, MA, USA). Then, RT-qPCR assays were used to detect mtDNA levels (ND1) in the cytosol and nuclear DNA levels (β2M) in samples. The sequences of primers are listed in Supplementary Table 3.

### RNA extraction and quantitative RT-PCR

We extracted total RNA from HUVEC by the TRIzol reagent (R401, Vazyme, Jiangsu, China). The HiScript III RT SuperMix (R323, Vazyme) was used for the RNA’s reverse transcription. And SYBR qPCR Master Mix (Q711, Vazyme) was used to analyze the mRNA levels. The sequences of gene primers were listed in Supplementary Table 3.

### RNA sequencing

Total RNA was extracted from HUVEC, which in the absence and presence of C75 before LPS stimuli. Truseq RNA Library Prep Kit was used to construct the RNA libraries. Then we used Hisat2 (version 2.1.0) and RSEM (version 1.3.1) respectively to map or perform the clean data (reads) and gene expression analysis. DESeq2 was used to conduct differential expression analysis among different groups. |FC|>2 and FDR < 0.05 were determined as thresholds for differentially expressed genes (DEGs). The KEGG analysis was used to identify the most significant signaling pathways involved in C75-regulated genes (adjusted *P* < 0.05).

### Histological and Immunohistochemical staining

Paraffin-embedded mice lung tissue sections were stained with H&E for blinded histopathologic assessment. For IHC, the sections were stained with VCAM-1 antibody (1:500, ab134047, Abcam) at 4 °C overnight, followed by incubation with secondary antibody for 30 min at 37  °C, and finally visualized with a 3,3’-diaminobenzidine solution and counterstained with hematoxylin. The images were taken using a light microscope (BX50, Olympus).

### Confocal laser microscope assay

HUVEC were seeded on the glass (PEZGS0416, Millipore) or in special confocal petri dish. For mtDNA detected, cells were firstly incubated with Mito-Tracker Red CMXRos (C1049B, Beyotime, China) after indicated treatments for 1 h at 37 °C. Then, for all these experiments, we used 1% paraformaldehyde and 0.25% Triton X-100 respectively to fix and permeabilize HUVEC. Next, we used 3% BSA to block and incubated with NF-κB p65 antibody (10745-1-AP, proteintech, China) at 4 °C overnight. Next day, the cells were incubated with secondary antibody (A32732 or A-11001, Thermo Fisher Scientific). The nuclei were stained with DAPI (D9542, Sigma–Aldrich). The images were taken using confocal laser microscope (DP73, Japan). All image analyses were performed by Fiji (Image J) and quantitative results from 8 cells for each condition are reported.

### Enzyme-linked immunosorbent assay

The concentrations of patients’ plasma sVCAM-1 and sE-selectin, and the concentrations of mice TF, TNF-α, and IL-6 were measured by using ELISA kits (MultiSciences Biotechnology, Hangzhou, China) following the instructions.

### Co-immunoprecipitation (Co-IP)

Cells were seeded at 10 cm dishes and were harvested at the confluent degree of 80-90%. And according to the instruction, we used the Co-IP Kit (B24011206, ACE, China) for subsequent experiments. Added 500 μL lysis buffer to each dishes for 1 h, and the lyses were centrifuged at 12,000 × *g* for 10 min at 4 °C. Then, collected the supernatant and detected the protein concentration by BCA. The supernatant was incubated with target antibody overnight at 4 °C. Subsequently, for immunoprecipitation, beads were added to supernatant to incubate with proteins overnight at 4 °C. Then proteins were eluted and denatured for further western blot assay.

### CoIP-MS analysis

Co-IP Kit (B24011206, ACE, China) was used for the sample preparation of mass spectrometry. The supernatant of HUVEC lysate was incubated with antibody against ETS1 (#14069, Cell Signaling Technology, USA) overnight at 4 °C. Next, magnetic beads were added into the mixture and again incubated at 4 °C overnight. The precipitation complex was washed to remove the non-specifically bound proteins. The magnetic beads-antigen-antibody complex samples were used for LC-MS/MS analysis (OE Biotech Co., Ltd, Shanghai). The nano-HPLC liquid phase system Easy-NLC1200 was used for separation, the dried polypeptide samples were first re-dissolved in Nano-HPLC Buffer A (liquid A was 0.1% formic acid-aqueous solution, and liquid B was 0.1% formic acid-acetonitrile solution). 100 μm × 20 mm (RP-C18, Thermo Inc.) with 100% liquid A equilibrium. The samples were then loaded by an automatic sampler and adsorbed to a Trap column, and separated on an Analysis column, 75 μm × 150 mm (rp-c18, thermo Inc.) at a flow rate of 300 nL/min. The samples were cleaned by mobile phase gradient with blank solvent for 30 min. The hydrolysates were separated by capillary high-performance liquid chromatography and analyzed by Q-Exactive mass spectrometry (Thermo Scientific).

### Molecular docking analysis

ETS1 (P14921-5) and VDAC1 (P21796) were downloaded from UniProt. AlphaFold3 was used for docking of ETS1 and VDAC1. pLDDT and PAE scores were used for model quality assessment [74]. ipTM + iTM > 0.75 was considered high interaction between two proteins [75].

### GST pull-down analysis

According to the manufacture instruction of GST pull-down kit (IK-2004, Biolinkedin, Shanghai, China), 50 µg of GST-controlled protein (HY-P70270, MedChemExpress, NJ, USA) or GST-tagged VDAC1 protein (ab132481, abcam, USA) was immobilized in 50 µL of glutathione agarose beads and equilibrated before incubated at 4 °C overnight. Then, 50 µg of His-tagged ETS1 protein (HY-P70292, MedChemExpress, NJ, USA) was added and incubated with GST-VDAC1 or GST-controlled protein at 4 °C overnight. Finally, the protein complexes were analyzed by using western blot to detect the direct interaction between ETS1 and VDAC1 protein.

### siRNA transfection

HUVEC were seeded at a density of 55–70% and transfected with small-interfering RNAs (GenePharma, Shanghai, China) for 48 h. The efficacy of transfection was validated by western blot analysis or RT-qPCR. HiPerFect (301,704, Qiagen, Germany) was used as the transfection reagent for HUVEC. The siRNA sequences are shown in Supplementary Table 3.

### Statistical analysis

We ensured all experiments were repeated independently at least three times. Data were presented as the mean ± SD. Statistical analyses and the experimental results were performed using SPSS (26.0, USA) and GraphPad (9.4.1, USA). Differences between groups were determined by two-tailed independent Student’s *t* test or one-way analysis of variance (ANOVA) followed with Bonferroni multiple comparison test. *P* < 0.05 was considered to be statistically significant.

## Supplementary information

original western blots

Supplementary Material

## Data Availability

The datasets used and/or analyzed during the study are available from the corresponding author upon reasonable request.
