# Supplementary material for: Fatty acid synthesis promotes mtDNA release via ETS1-mediated oligomerization of VDAC1 facilitating endothelial dysfunction in sepsis-induced lung injury
Source: Cell Death Differ. 2025 May 14;32(12):2177–92. doi: 10.1038/s41418-025-01524-5 (PMC12669790; doi:10.1038/s41418-025-01524-5)
Supplement: Supplementary file 1 — original western blots [file 41418_2025_1524_MOESM1_ESM.docx]

**Figure 2**

**I**


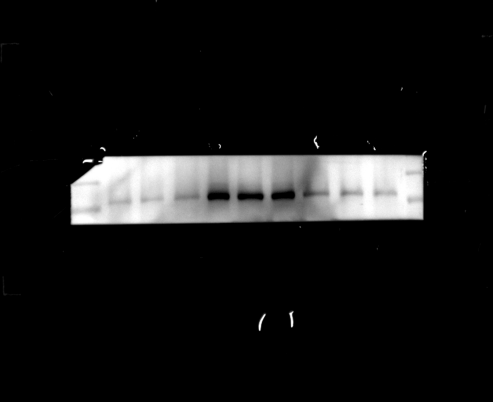
VCAM-1
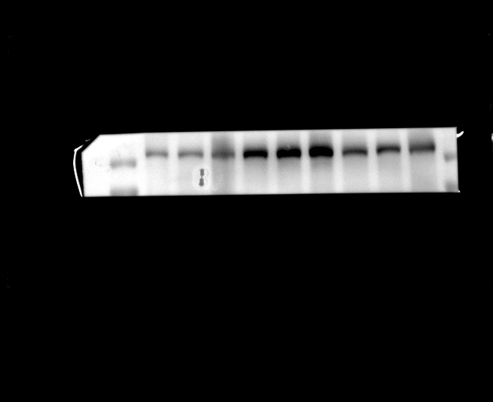
E-SEL


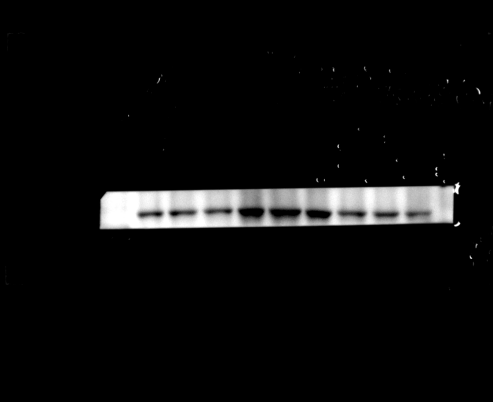
PAI-1
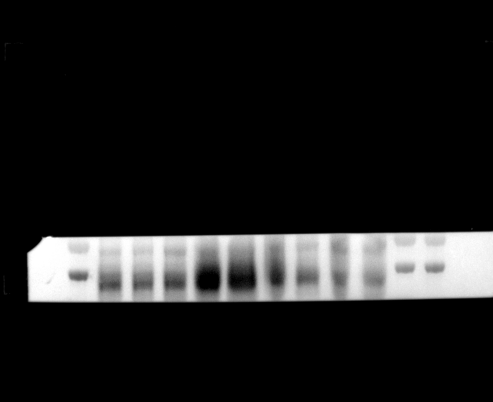
TF


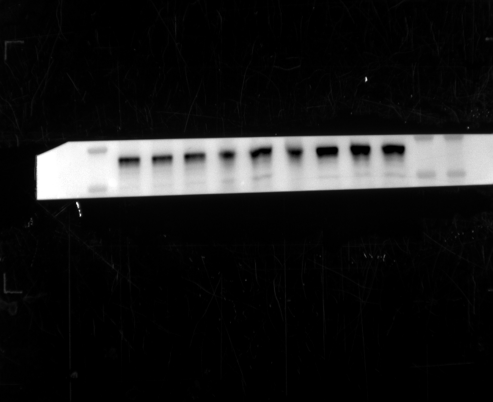
GAPDH

**Figure 3**

**A**

**
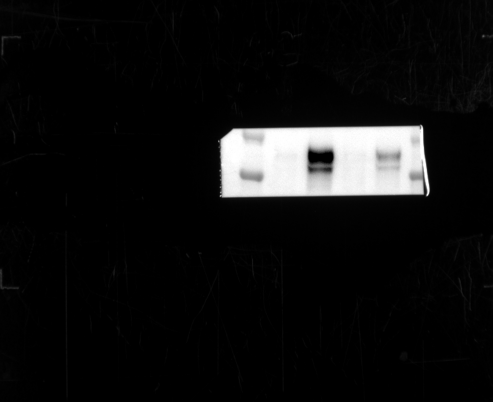
**VCAM-1
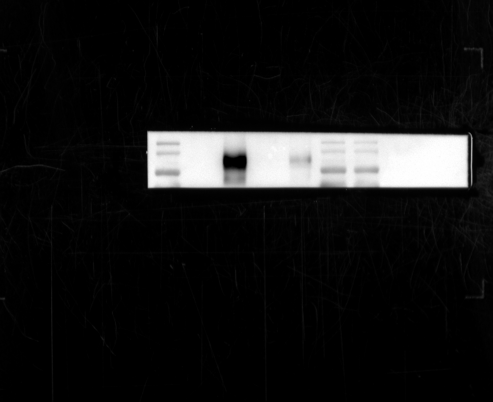
E-SEL


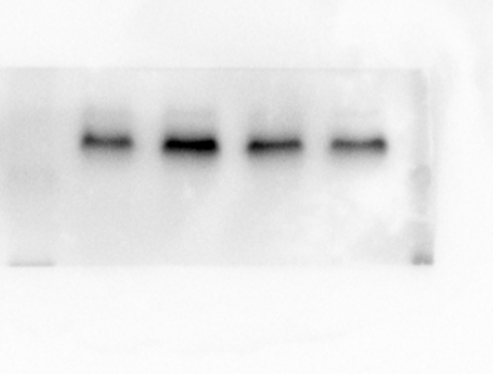
PAI-1
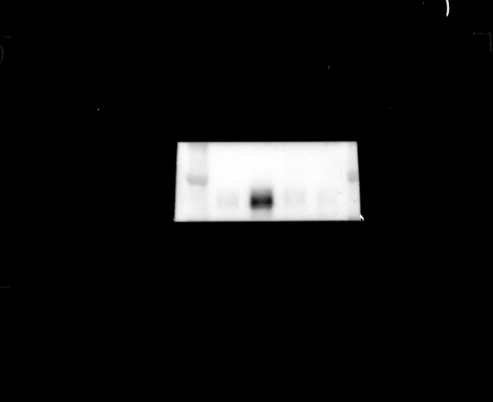
TF


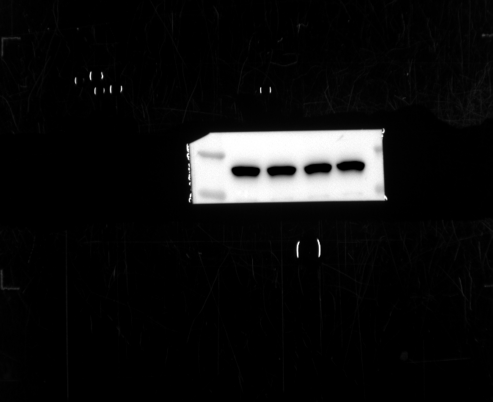
GAPDH

**J**


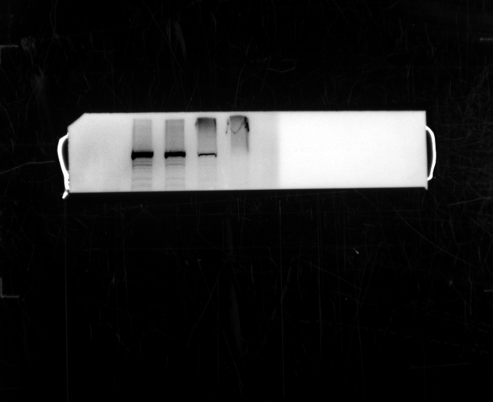
FASN
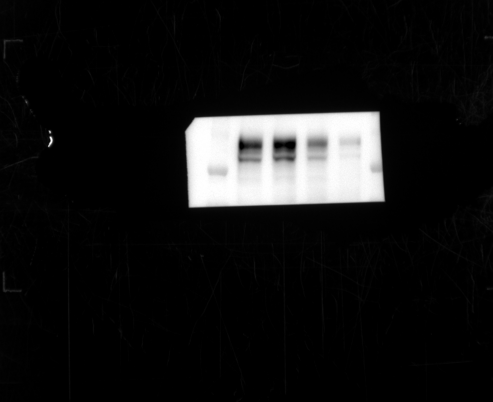
VCAM-1


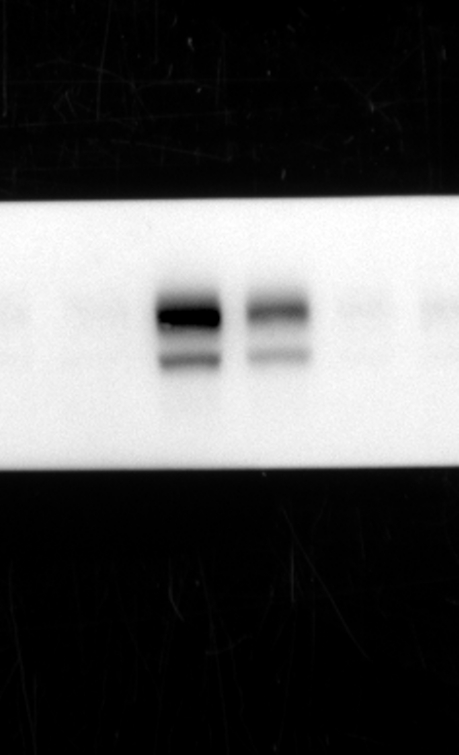
E-SEL **
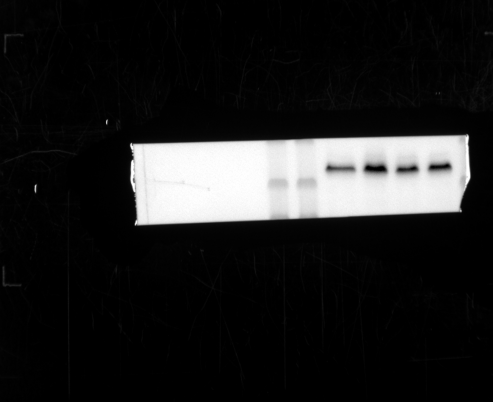
**PAI-1

**
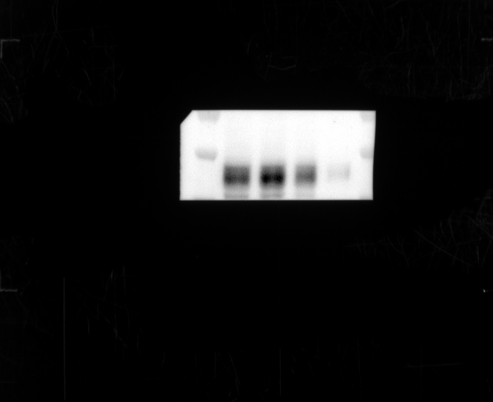
**TF
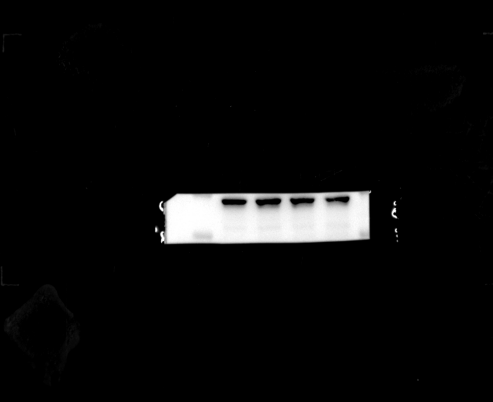
GAPDH

**L**


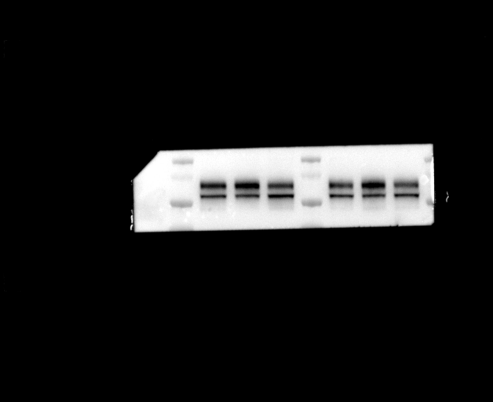
VCAM-1
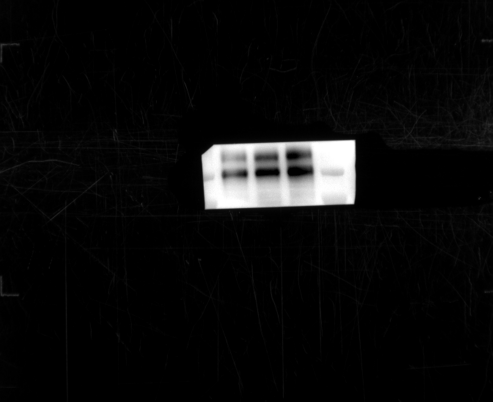
E-SEL


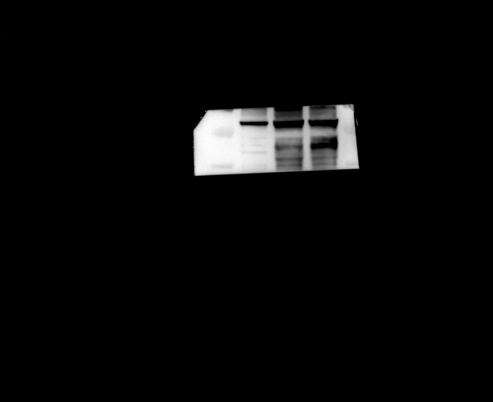
PAI-1
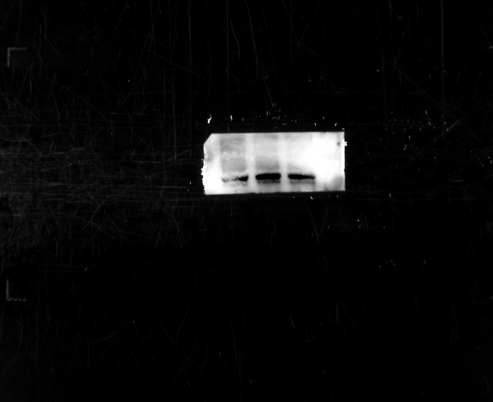
TF


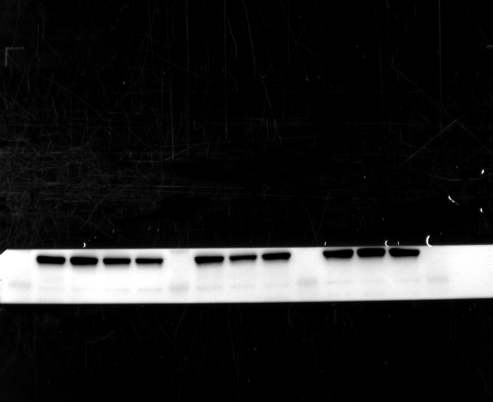
GAPDH

**Figure 5**

**B**

**
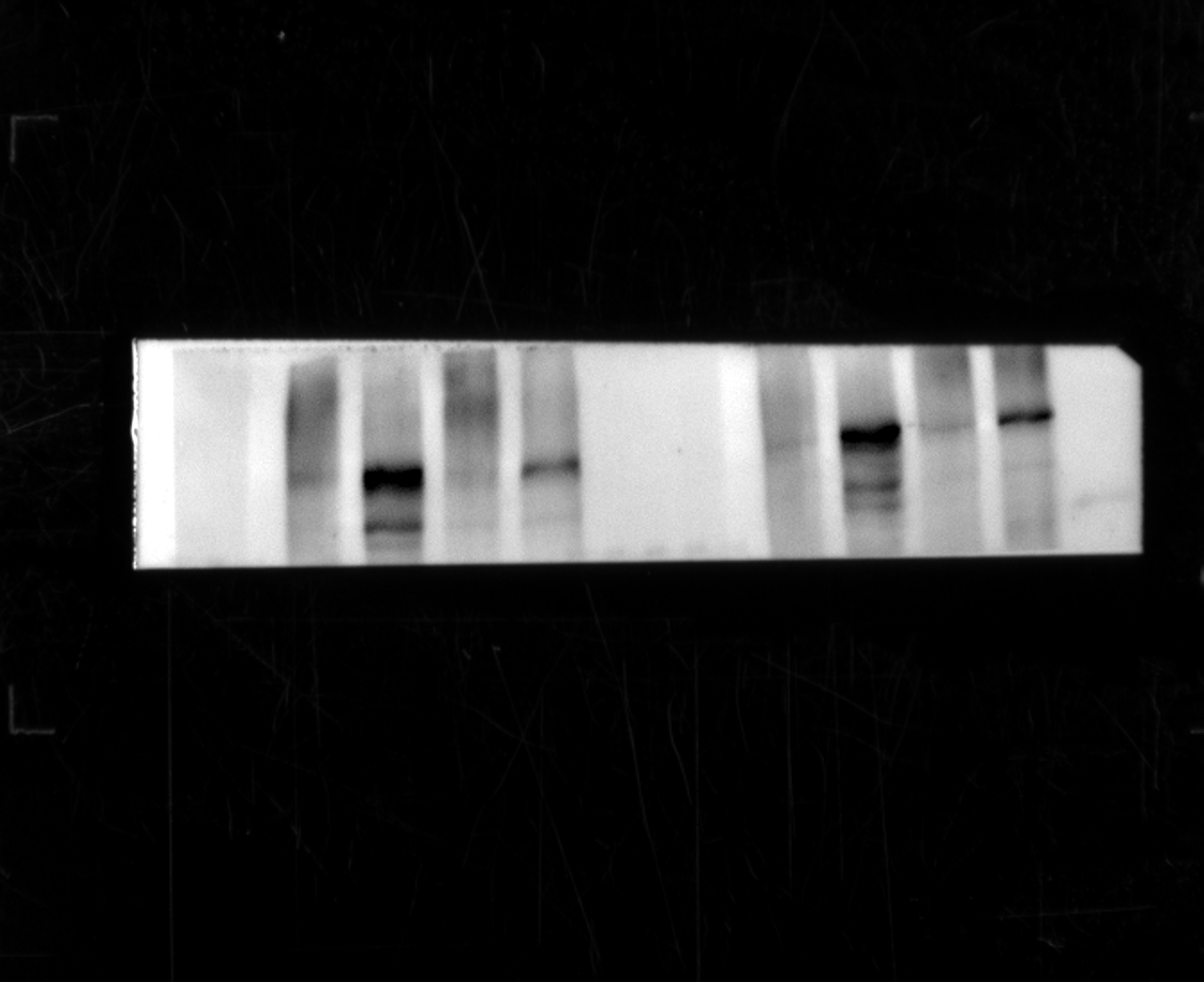
**cGAS
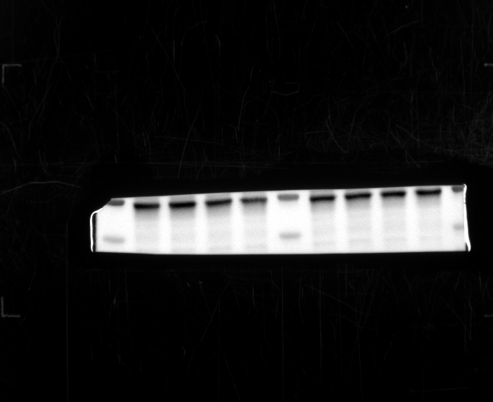
STING
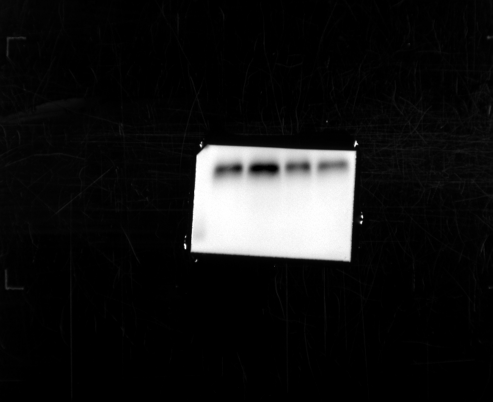
p-STING
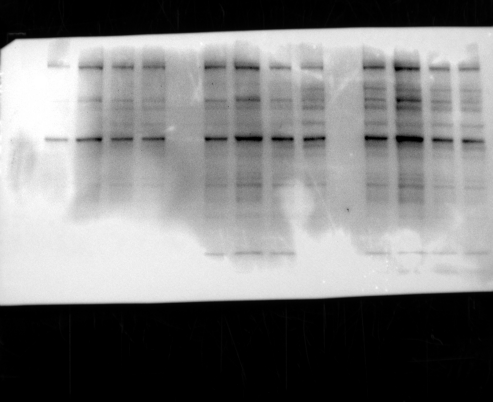
p-p65
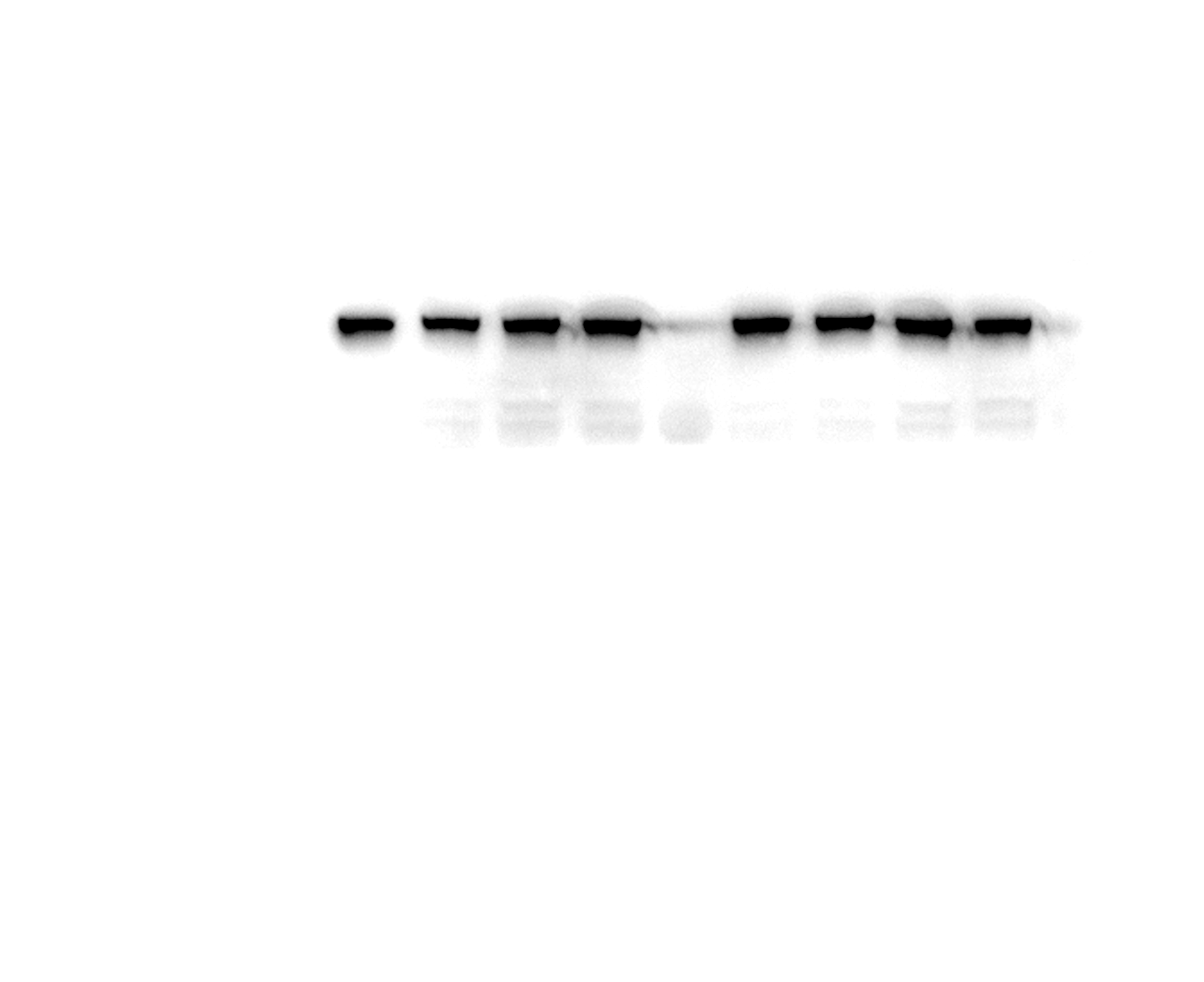
p65
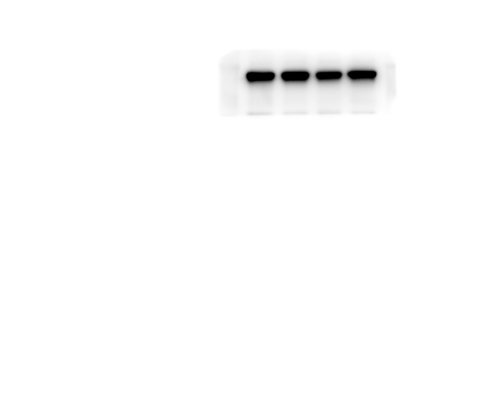
GAPDH

**F**


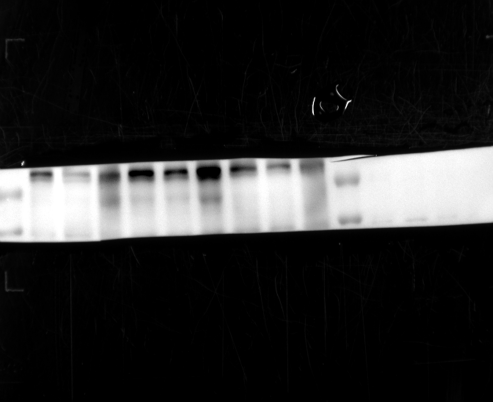
cGAS
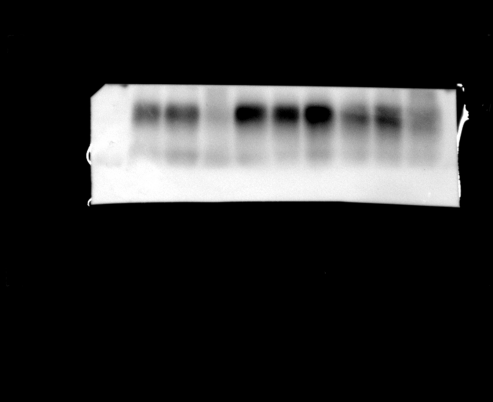
p-STING
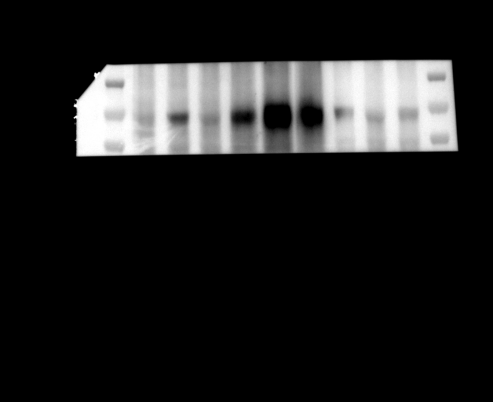
p-p65
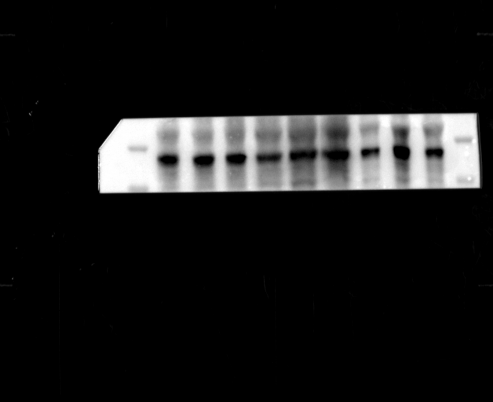
GAPDH

**Figure 6**

**A**


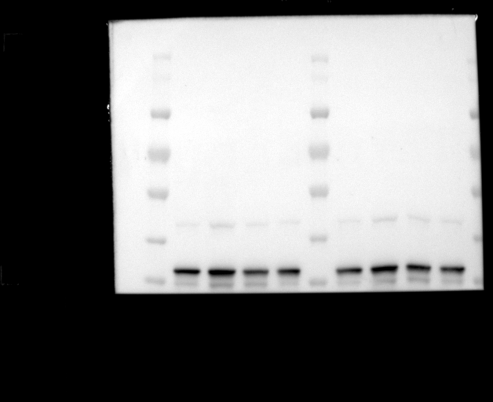
VDAC1-Monomer


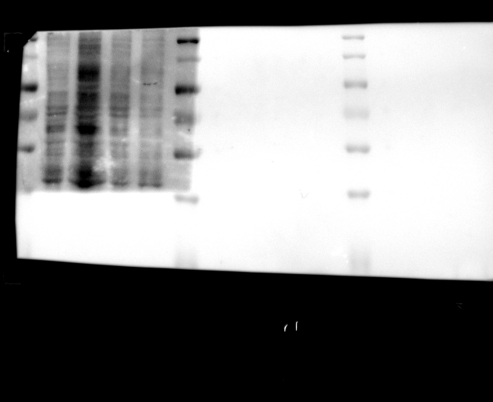
VDAC1-Oligomer
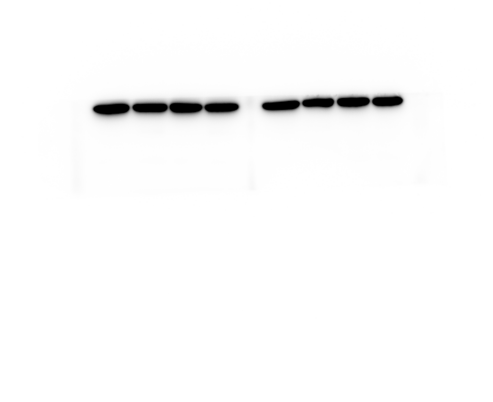
GAPDH

**C**


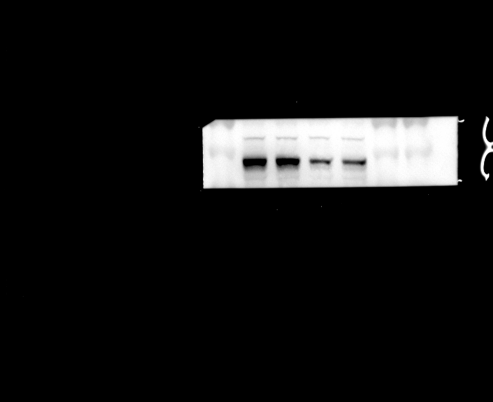
VDAC1-Monomer


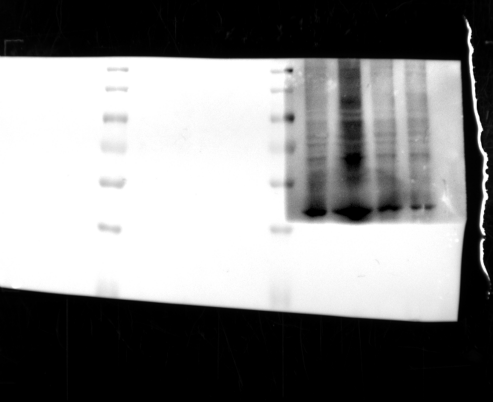
VDAC1-Oligomer
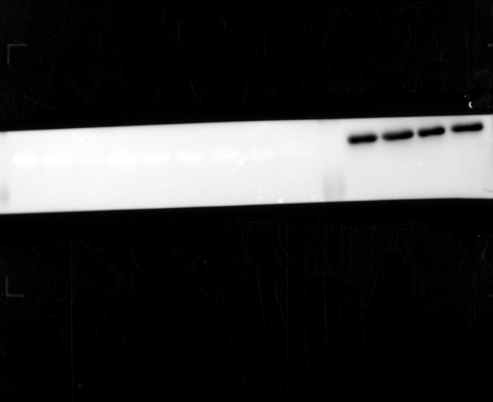
GAPDH

**E**


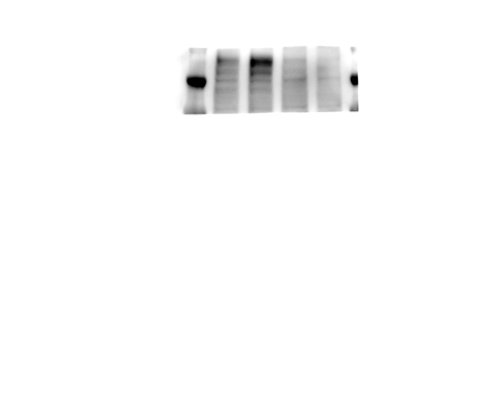
VCAM-1
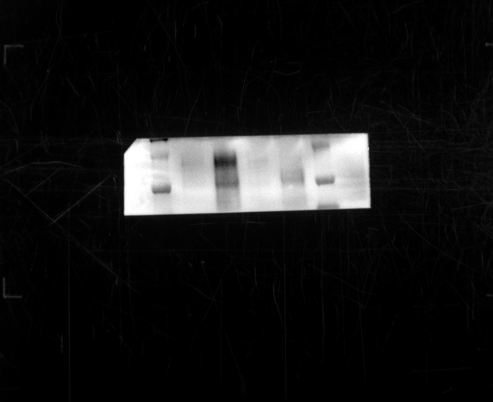
E-SEL
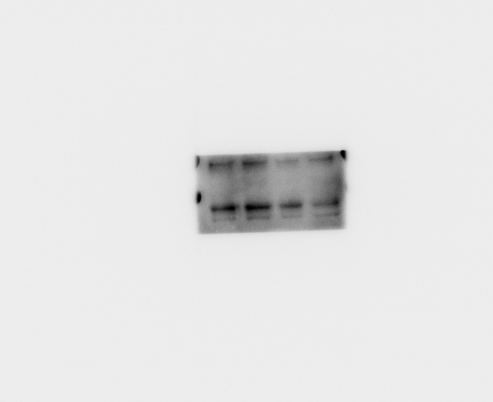
PAI-1
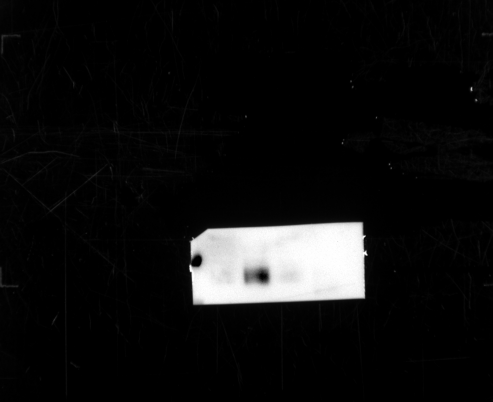
TF
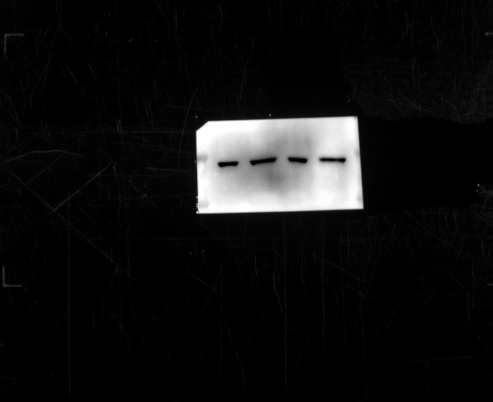
GAPDH

**G**

**
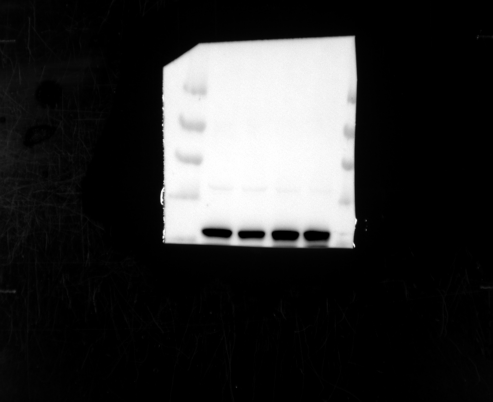
**VDAC1-Monomer

**
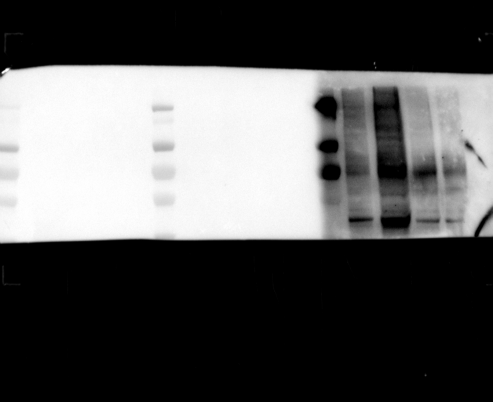
**VDAC1-Oligomer**
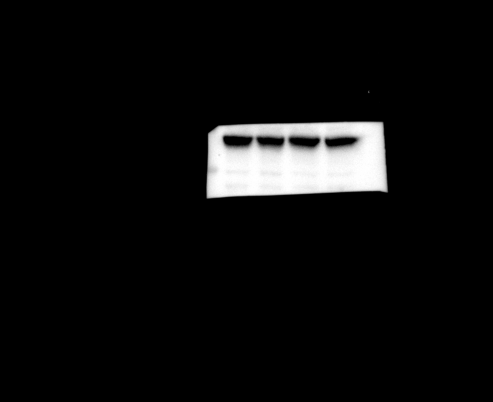
**GAPDH

**Figure 7**

**B**


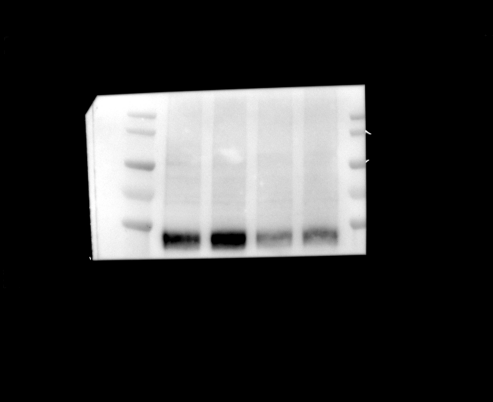
ETS1
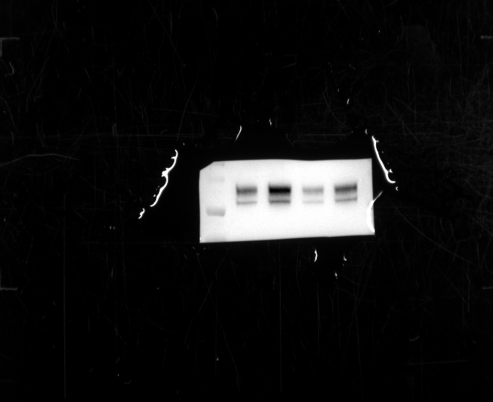
VCAM-1


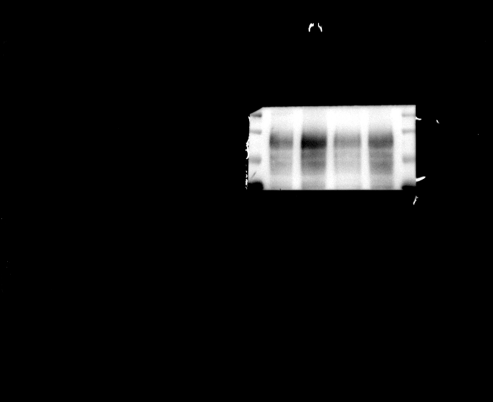
E-SEL
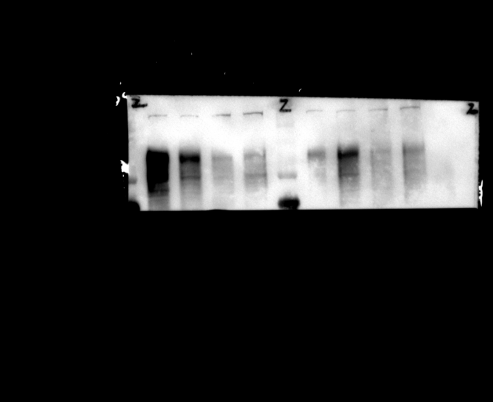
PAI-1


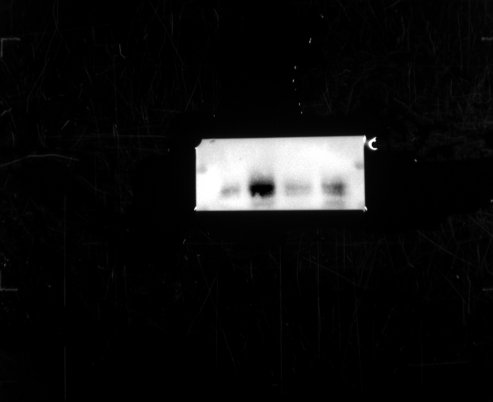
TF
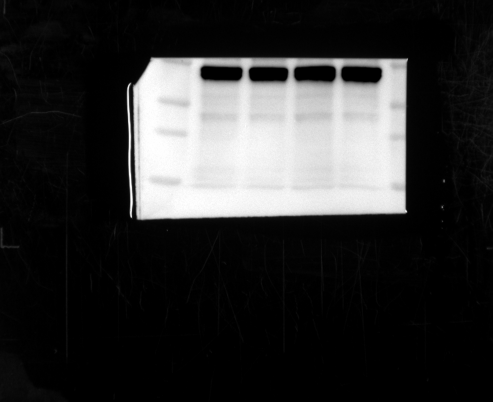
GAPDH

**G**

**
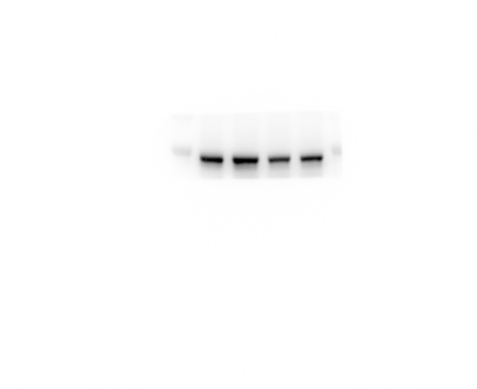
ETS1
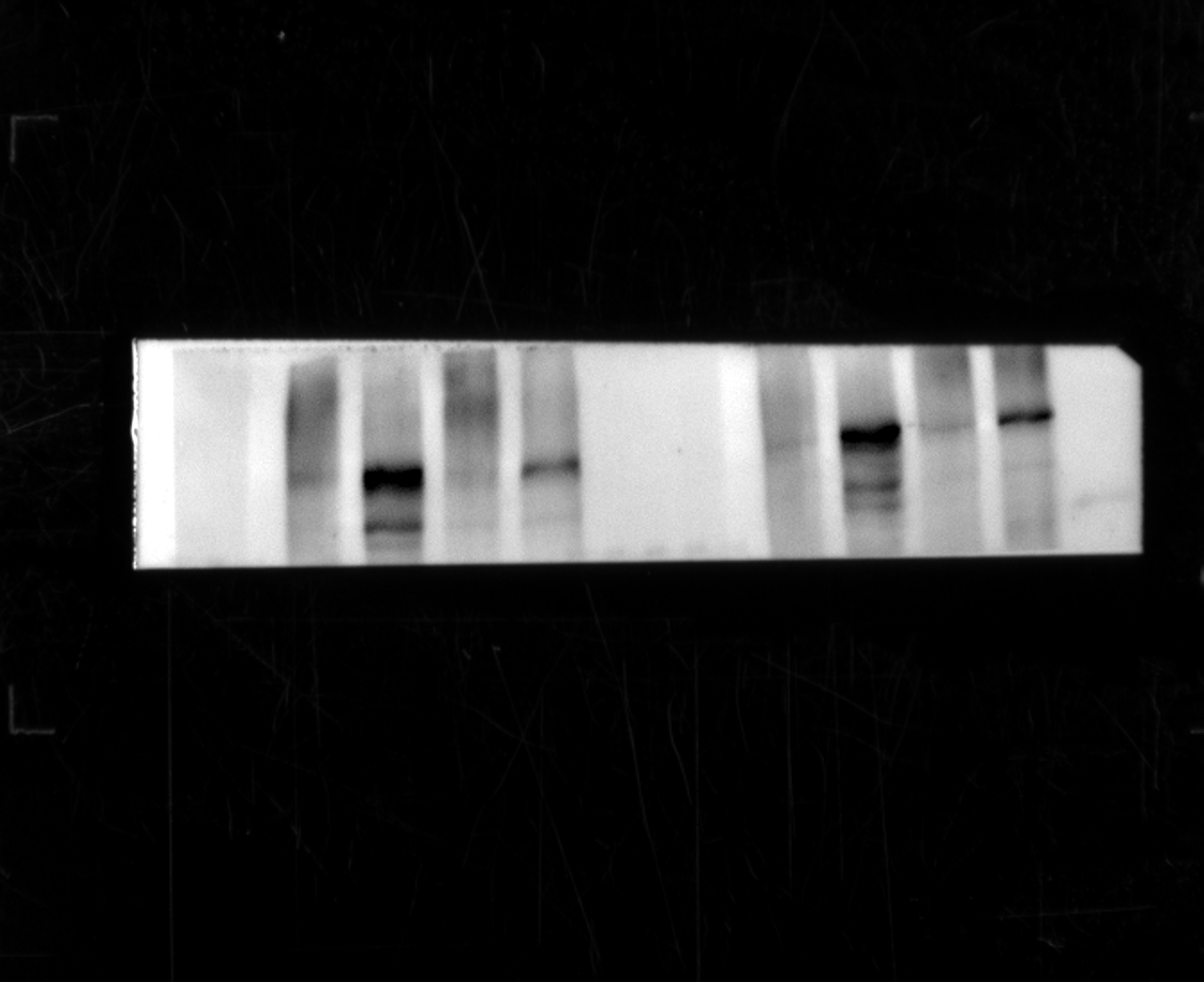
cGAS
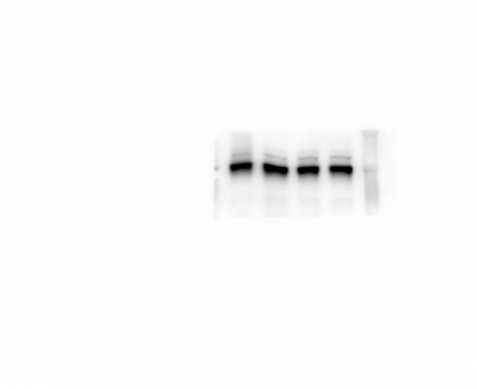
STING
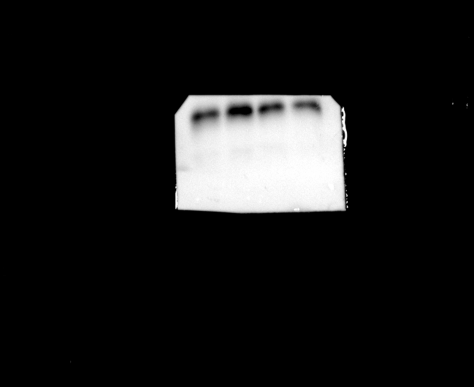
p-STING
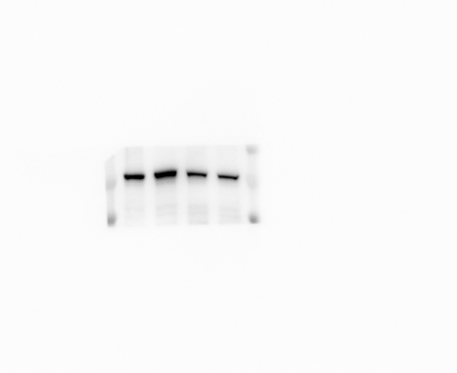
p-p65
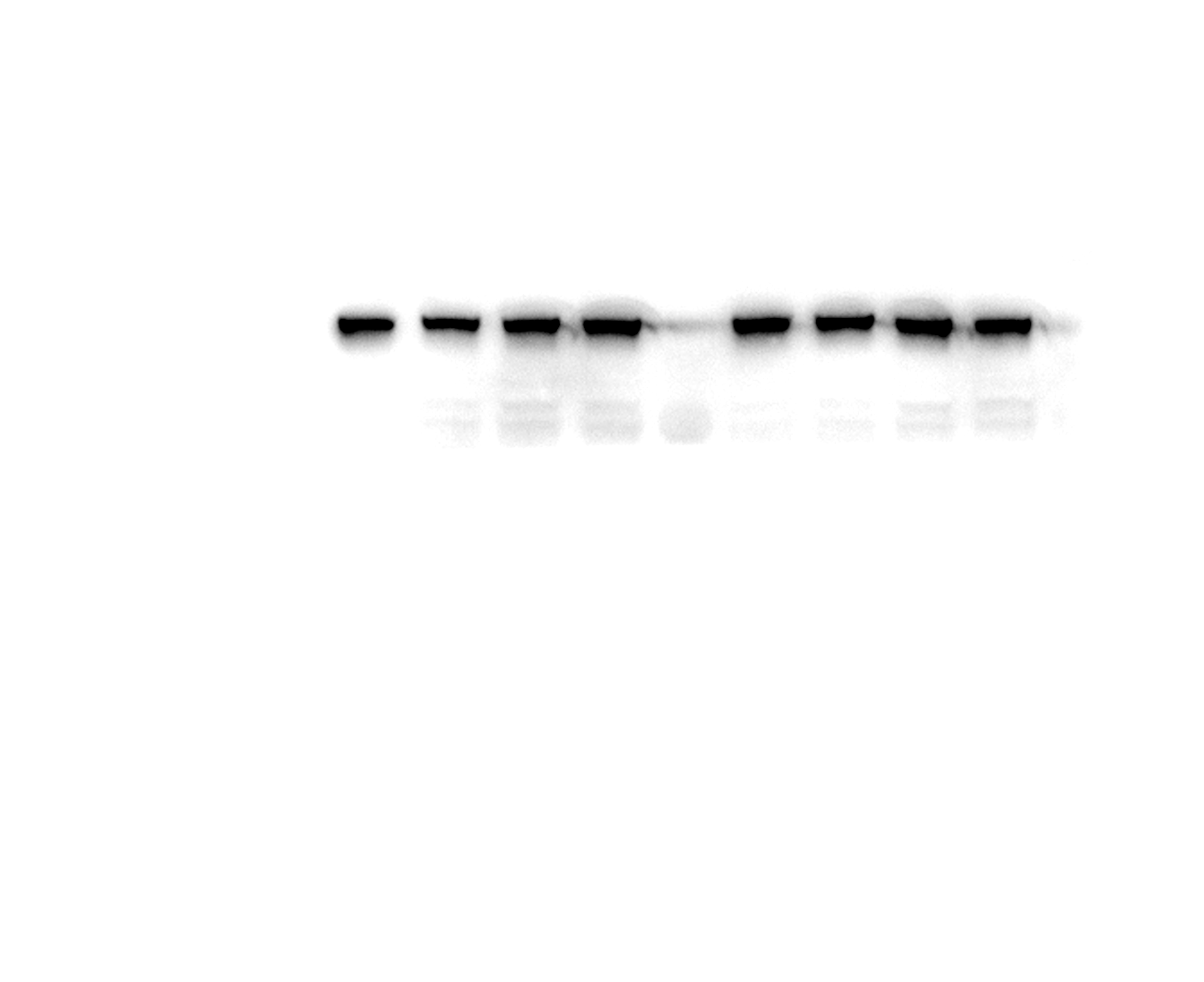
p65
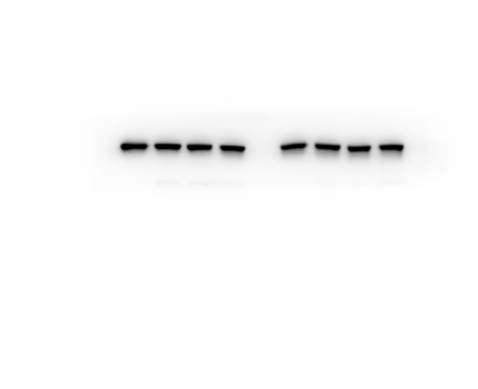
GAPDH**

**Figure 8**

**C**

**
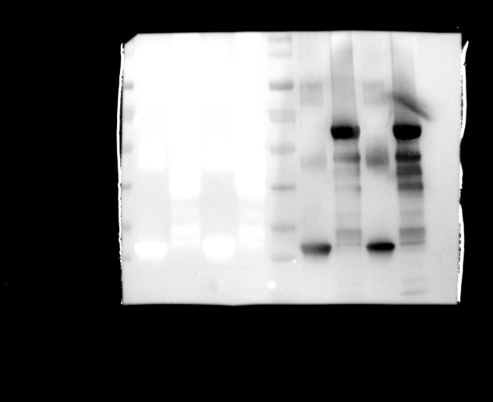
GST
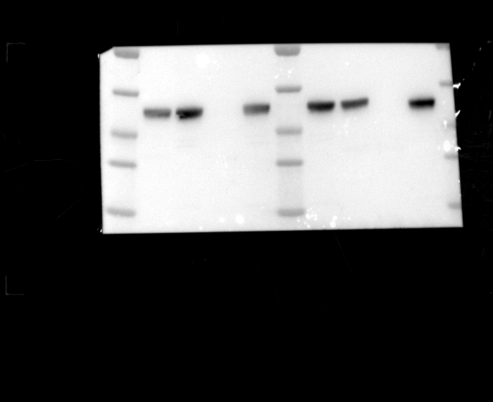
HIS**

**D**


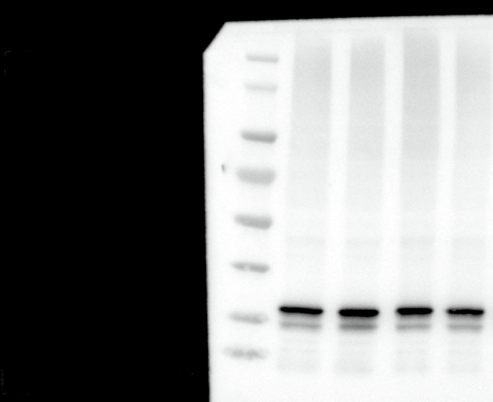
VDAC1-Monomer


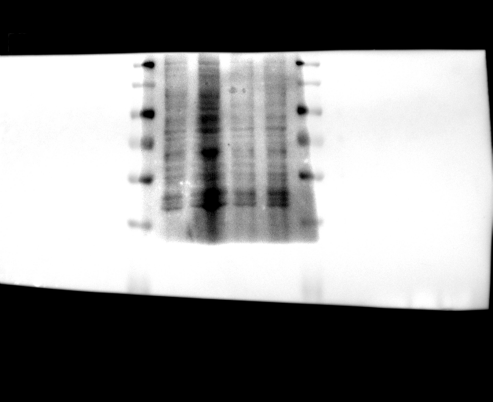
VDAC1-Oligomer
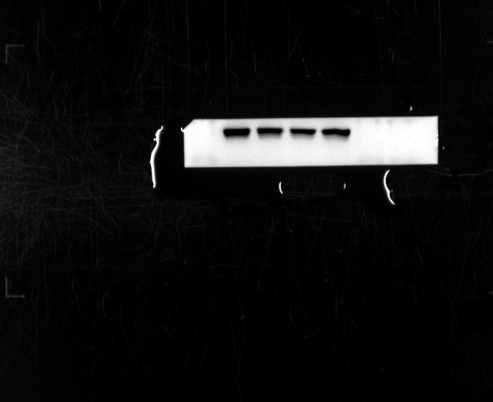
GAPDH

**G**


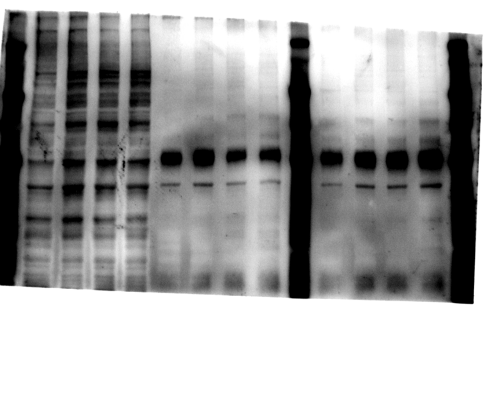
IP-VDAC1
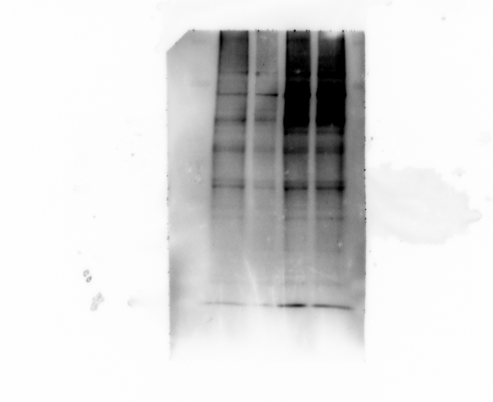
IP-Ubi


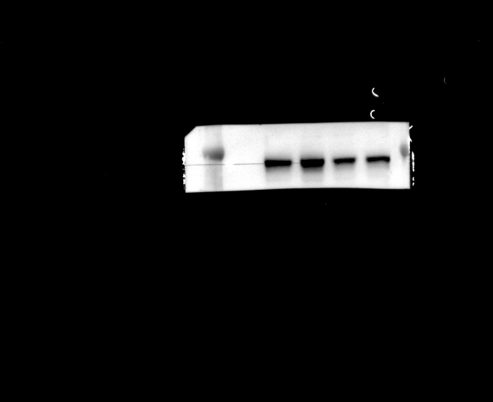
input-ETS1
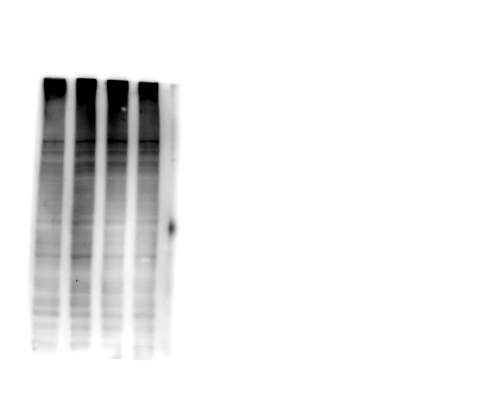
input-Ubi
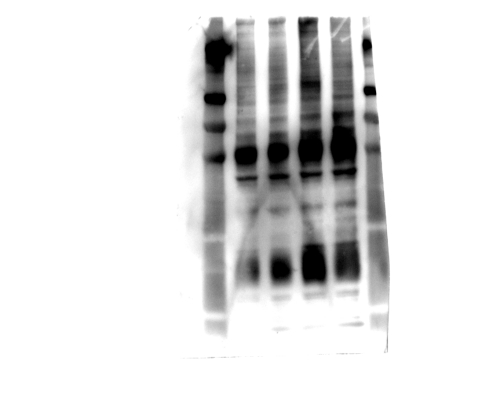
VDAC1-Monomer
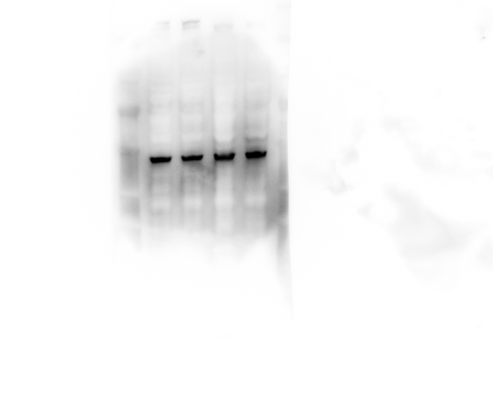
GAPDH

**H**


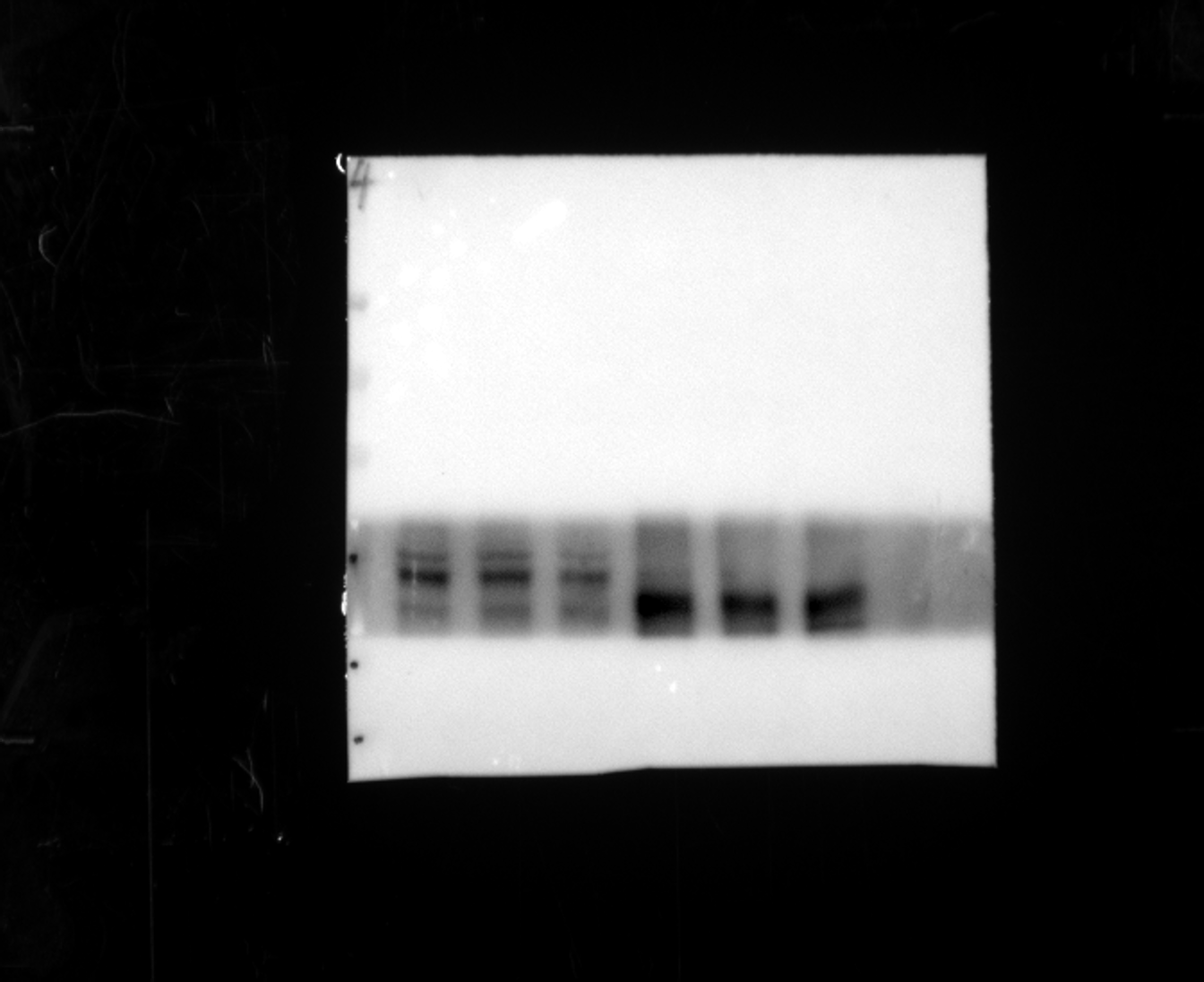
IP-VADC1
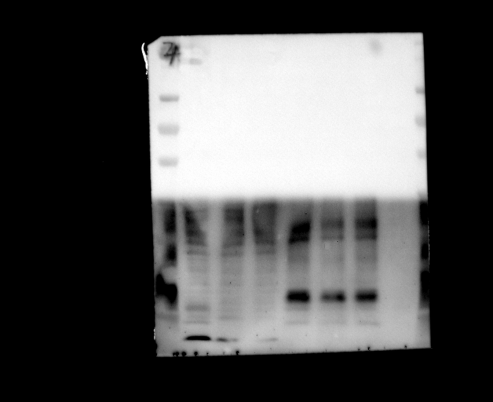
IP-UBi
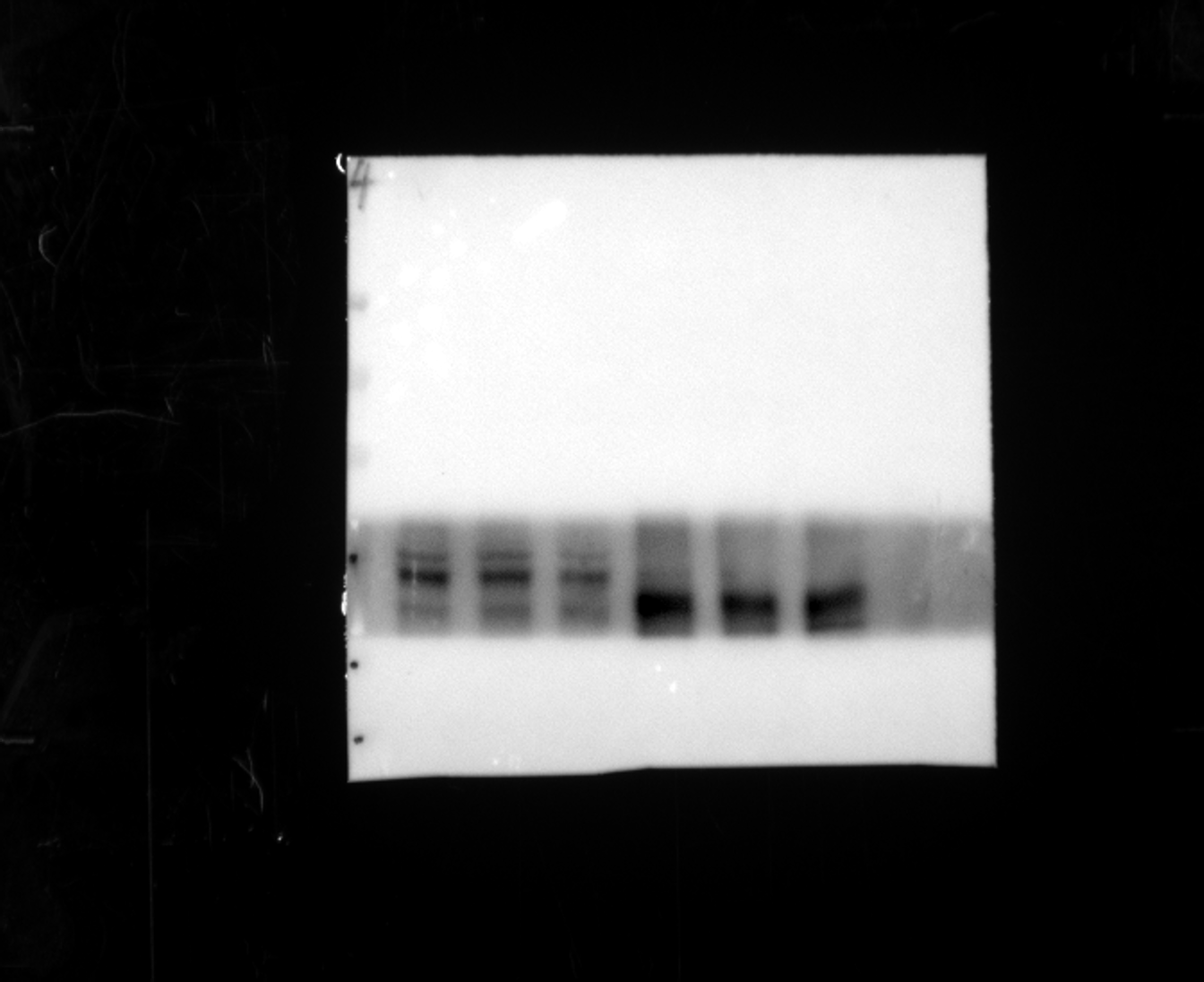
input-VDAC1
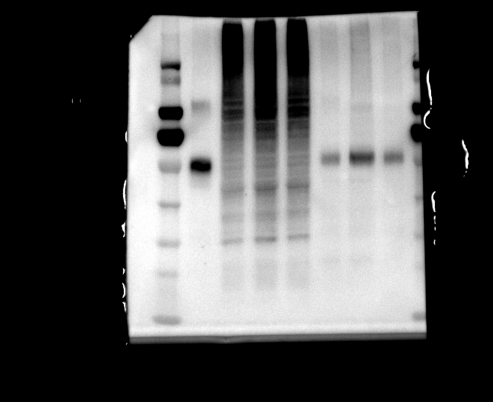
input-Ubi
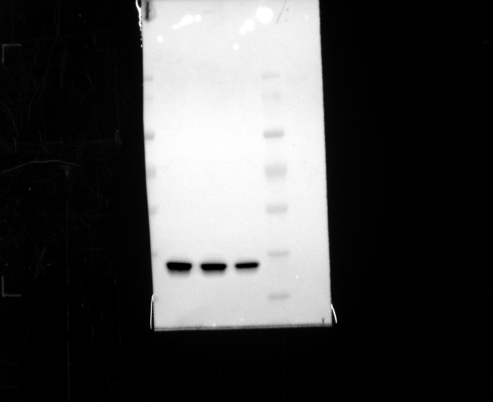
GAPDH

**Supplementary fig.1**

**B**

**
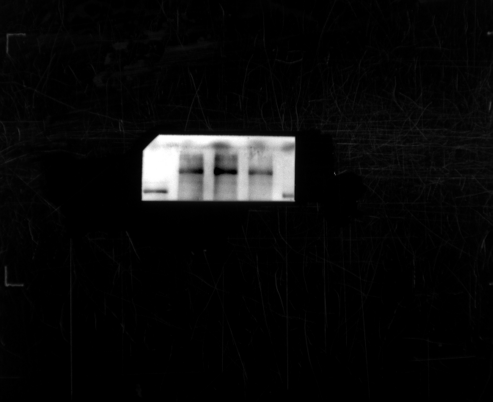
**FASN**
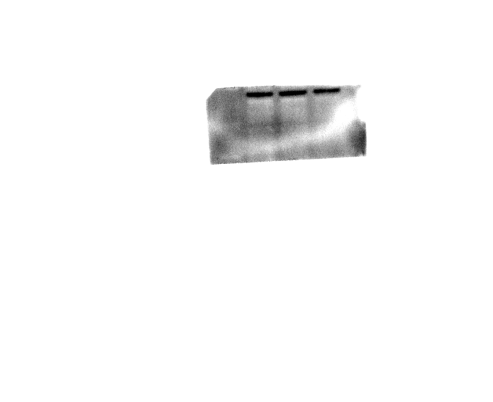
**GAPDH

**D**


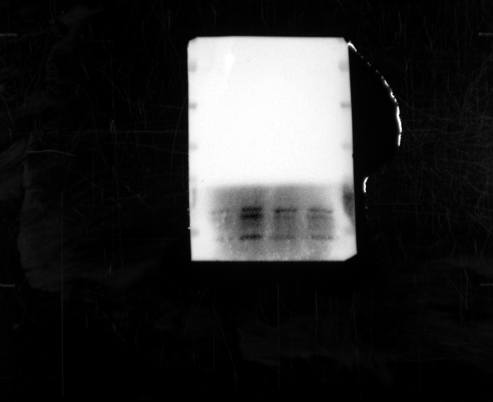
c-GSDMD
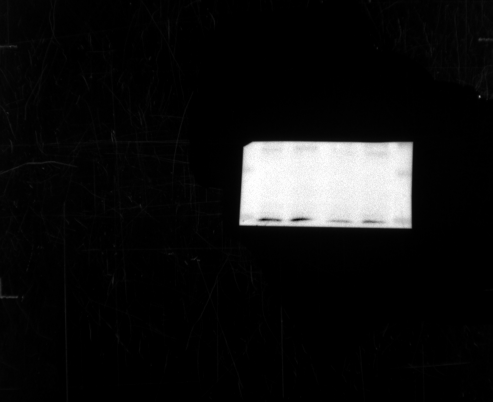
c-caspase1
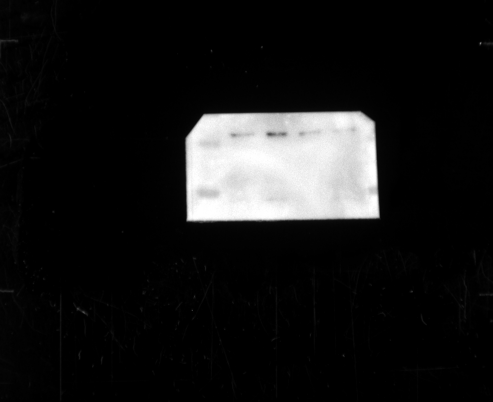
IL-1B
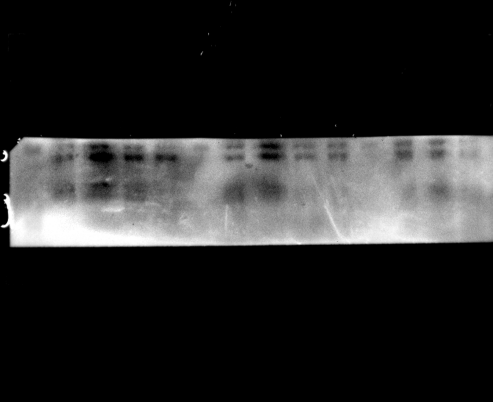
caspase4


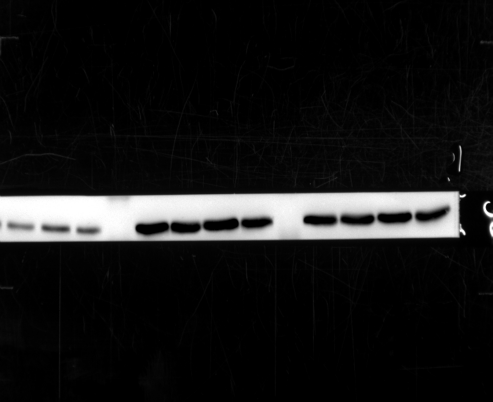
GAPDH

**F**

**
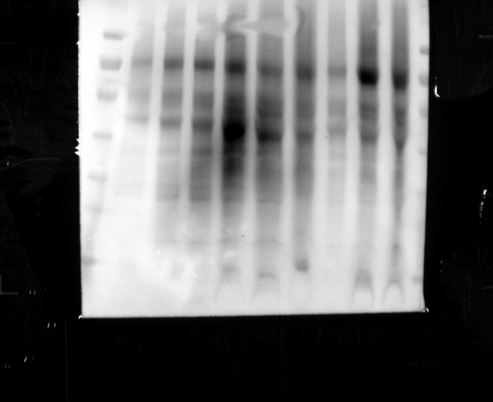
**c-caspase1
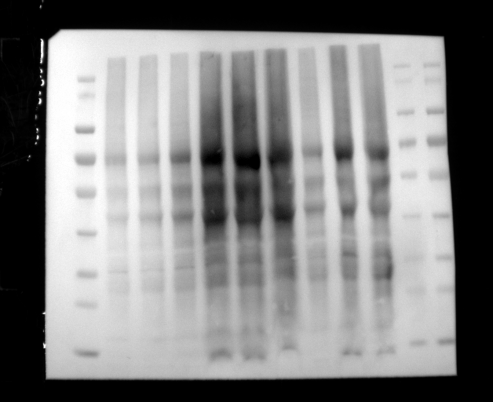
c-GSDMD
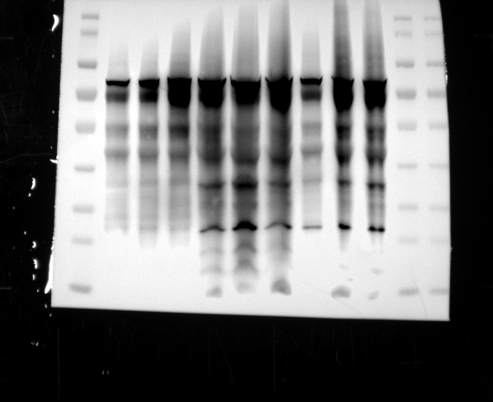
IL-1B
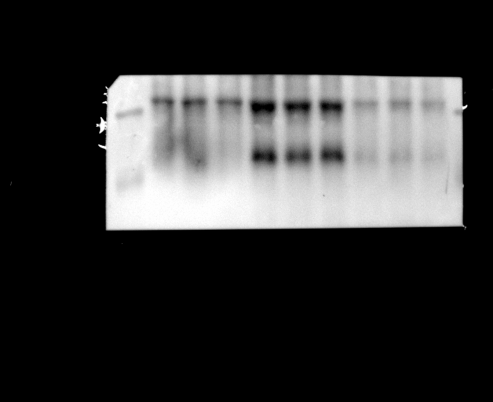
caspase11


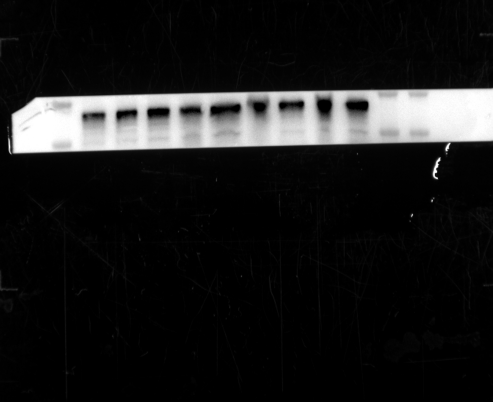
GAPDH

**Supplementary fig.2**

**A**


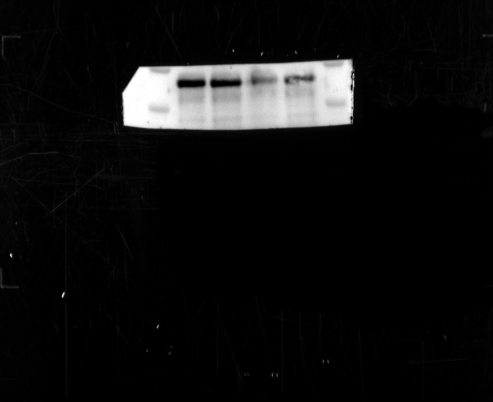
VDAC1
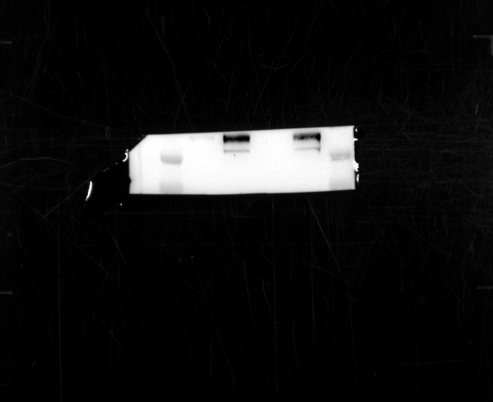
VCAM-1


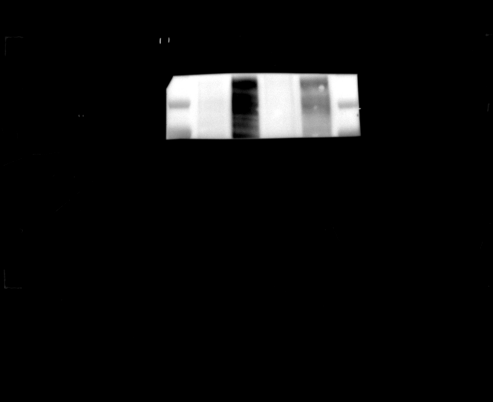
E-SEL
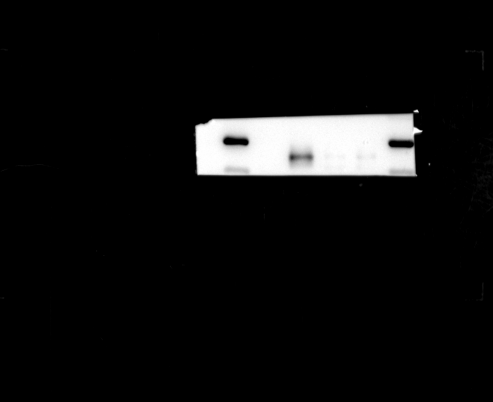
TF
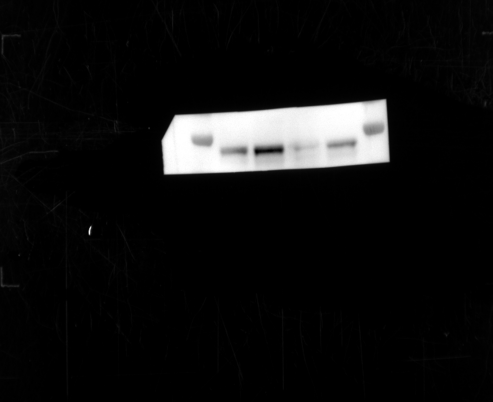
PAI-1
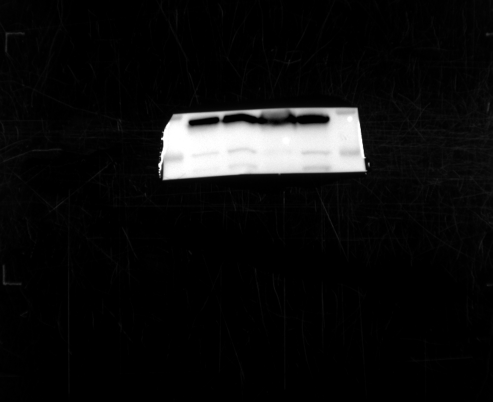
GAPDH

**Supplementary fig.3**

**A**

**
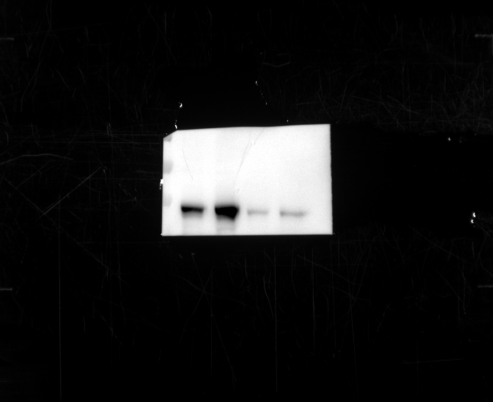
**ETS1 **
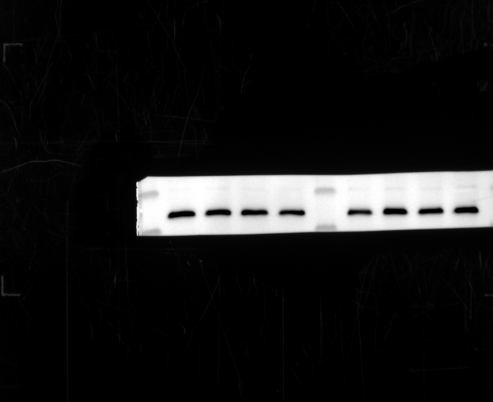
**GAPDH

**K**


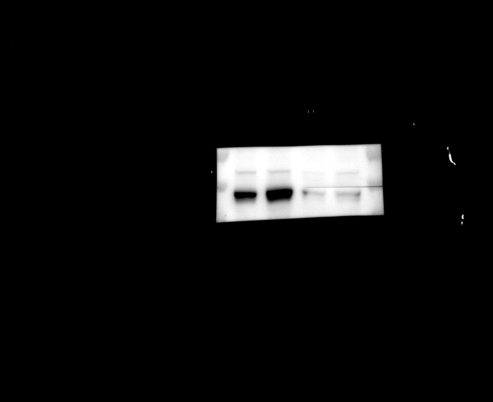
ETS1
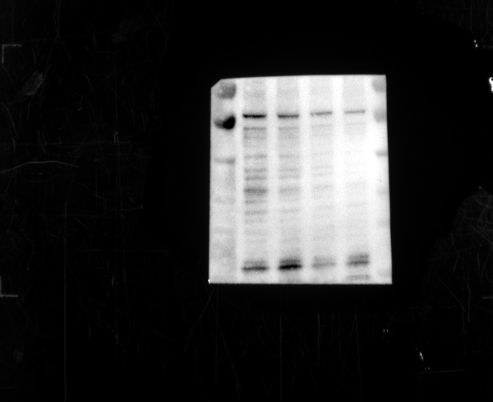
c-GSDMD
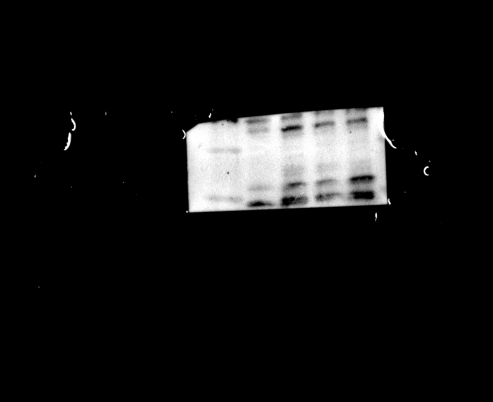
c-caspase1
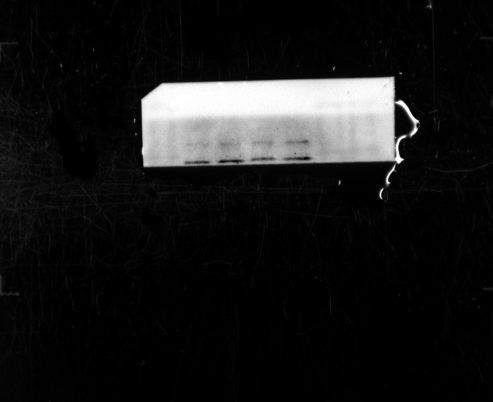
IL-1B
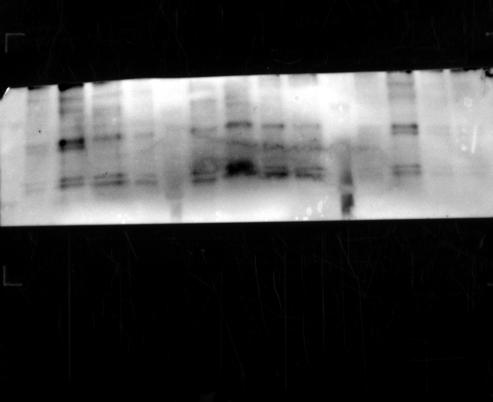
caspase4
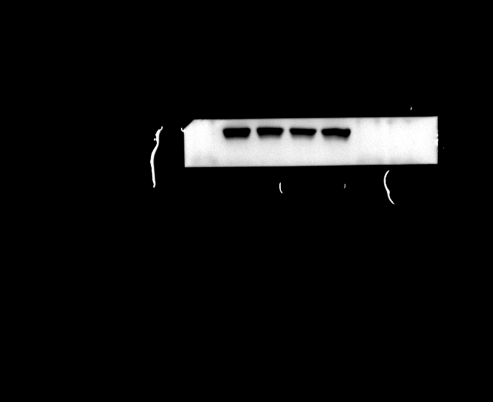
GAPDH

**Supplementary fig.4**

**A**

IP-ETS1 IP-VDAC1

input-ETS1 input-VDAC1

input-GAPDH

**B**

IP-VDAC1IP-UbiETS1 input-Ubi

input-VDAC1GAPDH
