# Supplementary material for: Fatty acid synthesis promotes mtDNA release via ETS1-mediated oligomerization of VDAC1 facilitating endothelial dysfunction in sepsis-induced lung injury
Source: Cell Death Differ. 2025 May 14;32(12):2177–92. doi: 10.1038/s41418-025-01524-5 (PMC12669790; doi:10.1038/s41418-025-01524-5)
Supplement: Supplementary file 2 — Supplementary Material [file 41418_2025_1524_MOESM2_ESM.pdf]

Fatty acid synthesis promotes mtDNA release via ETS1-mediated oligomerization of VDAC1 facilitating endothelial dysfunction in sepsis-induced lung injury

The catalog of Supplementary Material

Supplementary Table 1. Characteristics of septic patients in our study. 2

Supplementary Table 2. Information of antibodies 3

Supplementary Table 3. Sequences for siRNA and primers 4

Supplementary Figure Legends 5

- Supplementary Fig.1 Inhibition of fatty acid synthesis blocked LPS-induced pyroptosis in ECs5
- Supplementary Fig.2 The deletion of VDAC1 abolished LPS-induced EC activation 6
- Supplementary Fig.3 The deletion of ETS1 inhibited EC activation induced by LPS 7
- Supplementary Fig.4 The overexpression of ETS1 inhibited VDAC1 ubiquitination 8

Supplementary Table 1. Characteristics of septic patients in our study.

Characteristics	healthy controls	septic patients
**Numbers**	10	18
**Age (year)**	58±13	64±16
**Gender, male/female**	5/5	12/6
**BMI (kg/cm^2)**	21±0.8	22±1.5
**Sites of Infection, N (%)**		
Lung	-	8(44.4)
Abdominal	-	4(22.2)
Blood	-	1(5.6)
Others	-	5(27.8)
**SOFA Score**	-	6(3,12)
**APACHE II Score**	-	11(5,24)
**Laboratory Tests**		
CRP (mg/L)	-	152.5±93.8
WBC (10^9/L)	-	16.5±9.3
PLT (10^9/L)	-	227.7±98.8
APTT (s)	-	32.4±8.7
PT (s)	-	15.1±3.8
TT (s)	-	15.7±2.2
D-dimer (mg/L)	-	5.8±4.3
Fg (g/L)	-	4.5±1.8
**Complications，N (%)**		
Acute respiratory failure	-	12(66.7)
Acute cardiac dysfunction	-	5(27.8)
Acute hepatic insufficiency	-	3(16.7)
Acute kidney injury	-	8(44.4)
Septic shock	-	4(22.2)
**Hospital mortality, N (%)**	-	3(16.7)

Supplementary Table 2. Information of antibodies

Antibody	Source	Identifier
VCAM-1	Abcam	Cat: ab134047
IL-1β	Abcam	Cat: ab254360
Fibrinogen	Abcam	Cat: ab92572
dsDNA	Abcam	Cat: ab27156
CD31	Abcam	Cat: ab182981
Caspase 11	Abcam	Cat: ab246496
FASN	Cell Signaling Technology	Cat: #3180
ETS1	Cell Signaling Technology	Cat: #14069
ubiquitin	Cell Signaling Technology	Cat: #3936
p-sting	Cell Signaling Technology	Cat: #72971
caspase 1	Cell Signaling Technology	Cat: #83383
GSDMD	Cell Signaling Technology	Cat: #39754
goat anti-rabbit Alexa Flour 555	Cell Signaling Technology	Cat: #4413
goat anti-mouse Alexa Flour 488	Cell Signaling Technology	Cat: #4408
E-selectin	proteintech	Cat: No. 20894-1-AP
VDAC1	proteintech	Cat: No. 55259-1-AP
sting	proteintech	Cat: No. 19851-1-AP
p65	proteintech	Cat: No. 10745-1-AP
p-p65	proteintech	Cat: No. 82335-1-RR
cGAS	proteintech	Cat: No. 26416-1-AP
PAI-1	proteintech	Cat: No. 13801-1-AP
GAPDH	proteintech	Cat: No. 60004-1-Ig
Anti-rabbit IgG HRP-linked Antibody	proteintech	Cat: No. SA00001-2
Anti-mouse IgG HRP-linked Antibody	proteintech	Cat: No. SA00001-1
TF	Santa Cruz Biotechnology	Cat: SC-374441
caspase 4	MCE	Cat: HY-P81251

Supplementary Table 3. Sequences for siRNA and primers

**siRNA**	**Sense 5‘-3’**	**anti-sense 5‘-3’**
siFASN	GGUCCUUCUACUACAAGCUTT	AGCUUGUAGUAGAAGGACCTT
siETS1	GCAUAGAGAGCUACGAUAGTT	CUAUCGUAGCUCUCUAUGCTT
siVDAC1	GGGCUAUGGAUUUGGCUUATT	UAAGCCAAAUCCAUAGCCCTT
**Gene name**	**Sense 5‘-3’**	**anti-sense 5‘-3’**
VCAM-1	ACCACATCTACGCTGACAATGAATCC	AACACTTGACTGTGATCGGCTTCC
ICAM-1	GTCACCTATGGCAACGACTCCTTC	AGTGTCTCCTGGCTCTGGTTCC
E-selectin	GAAGAGGTTCCTTCCTGCCAAGTG	CAGAGCCATTGAGCGTCCATCC
TF	CCGACGAGATTGTGAAGGATGTG	TGTCTCCAGGTAAGGTGTGAACTC
PAI-1	CCGCCGCCTCTTCCACAAATC	TAGGGCAGTTCCAGGATGTCGTAG
IL-6	TTGGCCTCTGTGAAGGAACA	TGGCATCTTGTGGTGAGACC
MCP-1	CCCCAGTCACCTGCTGTTAT	CAGATCTCCTTGGCCACAAT
ETS1	AGCTTCGACTCAGAGGACTATCCG	GGCAGCAGCAGGAATGACAGG
GAPDH	GGTGAAGGTCG GAGTCAACG	CAAAGTTGTCATGGATGGACC
ND1	CTGGTCAACCTCAACCTAGGCC	GTTTGGGCTACTGCTCGCAGTG
β2M	CTGGGTAGCTCTAAACAATGTATTCA	CATGTACTAACAAATGTCTAAAATGGT

Supplementary Figure Legends

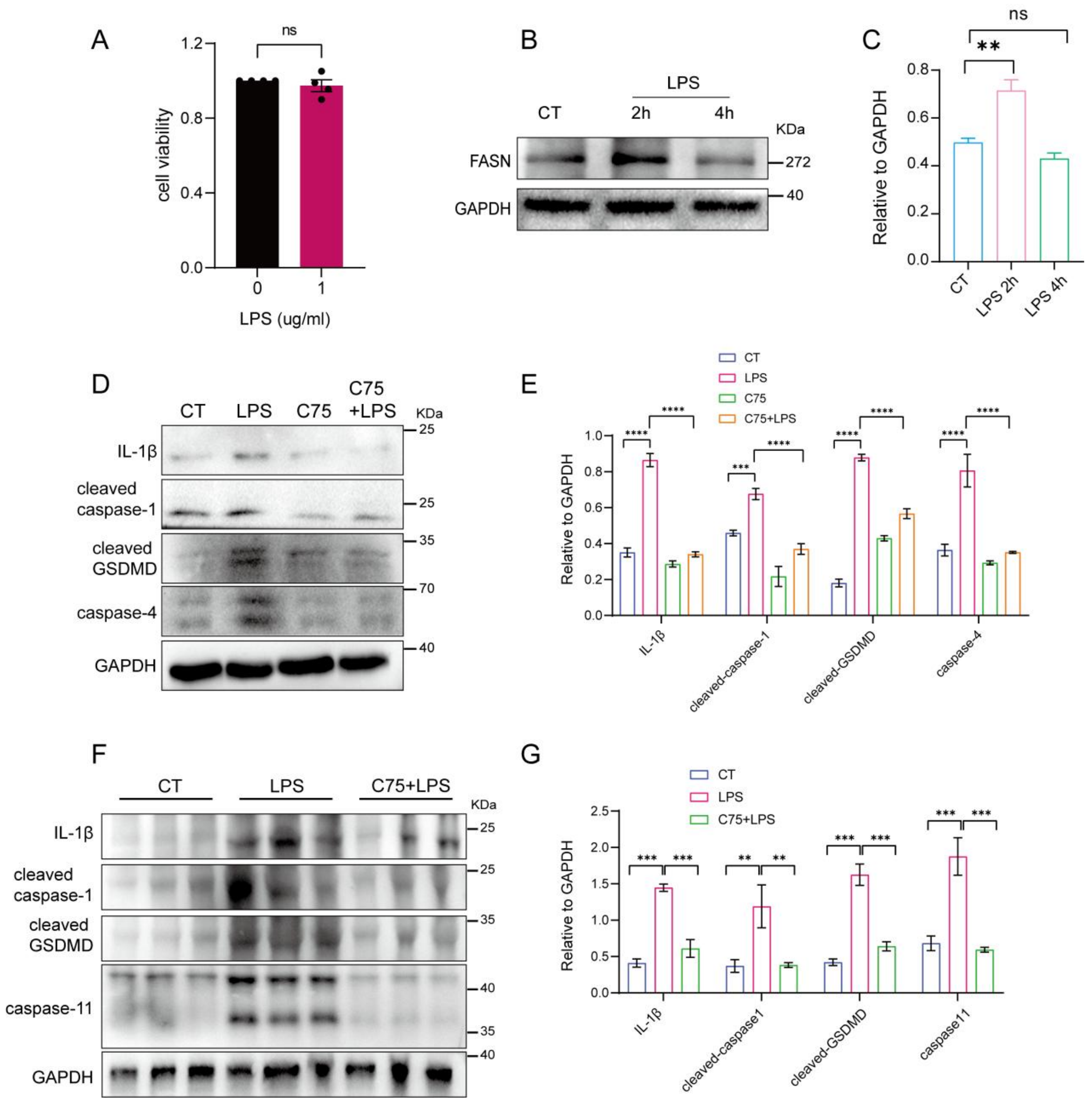

Supplementary Fig.1 Inhibition of fatty acid synthesis blocked LPS-induced pyroptosis in ECs

(A) The cell viability of HUVEC after 1 μg/ml LPS stimuli for 4 h by using CCK8 (n = 4). **(B-C)** Representative immunoblots of FASN with LPS stimuli (10 μM) for 2 h and 4 h (n = 3). **(D-E)** Representative immunoblots of cleaved IL-1β, cleaved caspase 1, cleaved GSDMD and caspase 4 with C75 pretreated before LPS stimuli in HUVEC. **(F-G)** Representative immunoblots of cleaved IL-1β, cleaved caspase 1, cleaved GSDMD and caspase 11 with C75 pretreated before LPS stimuli in mice (n = 3). All data were expressed as the mean ± SD. Unpaired t-test was used for the comparison between two groups. Comparison among three or more groups was analyzed by one-way ANOVA. ns, no significance, *$p < 0.05$, **$p < 0.01$, ***$p < 0.001$, and ****$p < 0.0001$.

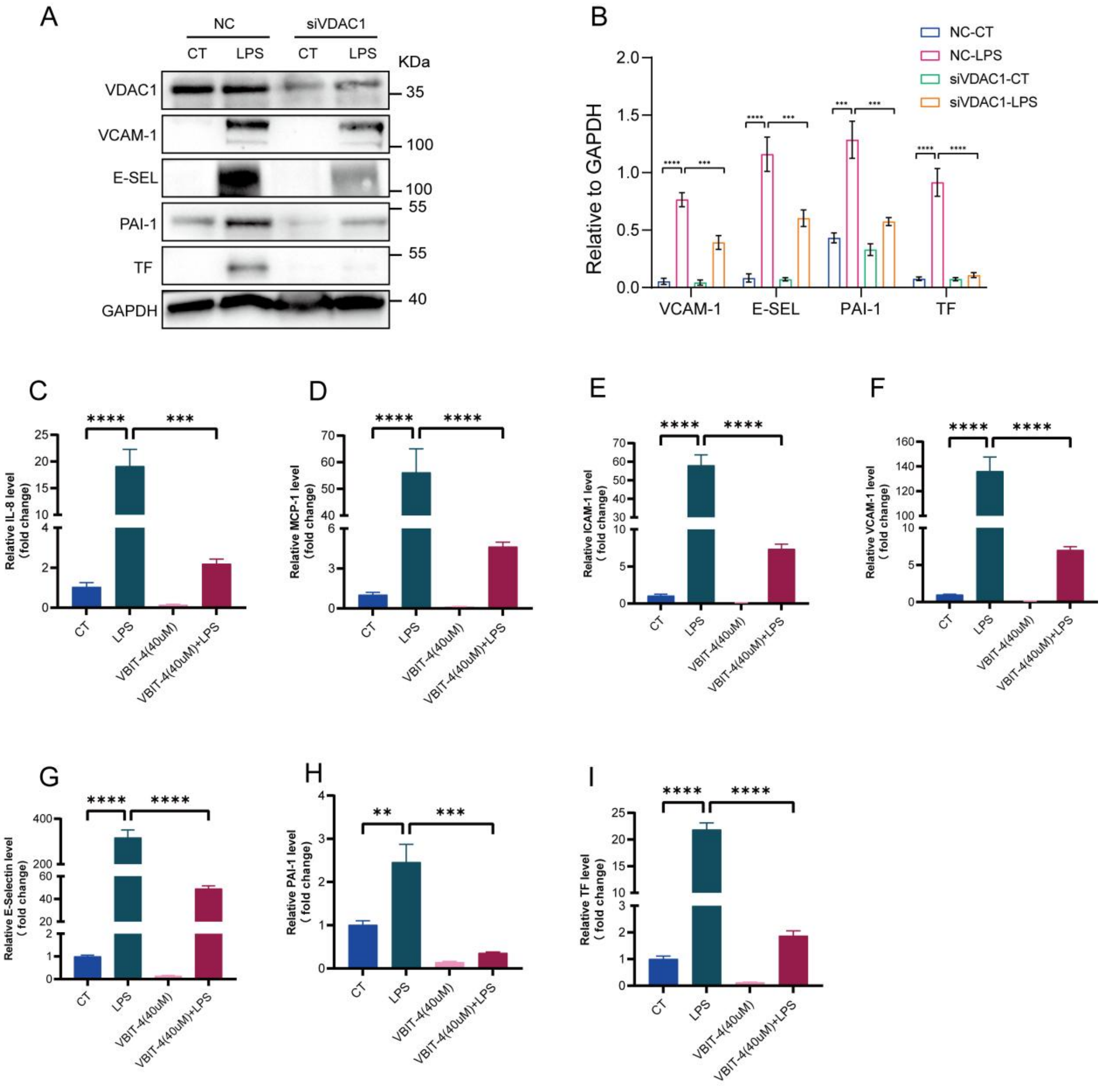

Supplementary Fig.2 The deletion of VDAC1 abolished LPS-induced EC activation

(A-B) HUVEC were transfected with siVDAC1 before LPS stimuli for 4 h. Expression levels of VDAC1, VCAM-1, E-selectin, PAI-1, and TF were analyzed by western blot (n = 3). **(C-I)** The mRNA levels of IL-8, MCP-1, ICAM-1, VCAM-1, E-selectin, PAI-1 and TF were detected by RT-qPCR (n = 3). All data were expressed as the mean ± SD. Comparison among three or more groups was analyzed by one-way ANOVA. ns, no significance, *p < 0.05, **p < 0.01, ***p < 0.001, and ****p < 0.0001.

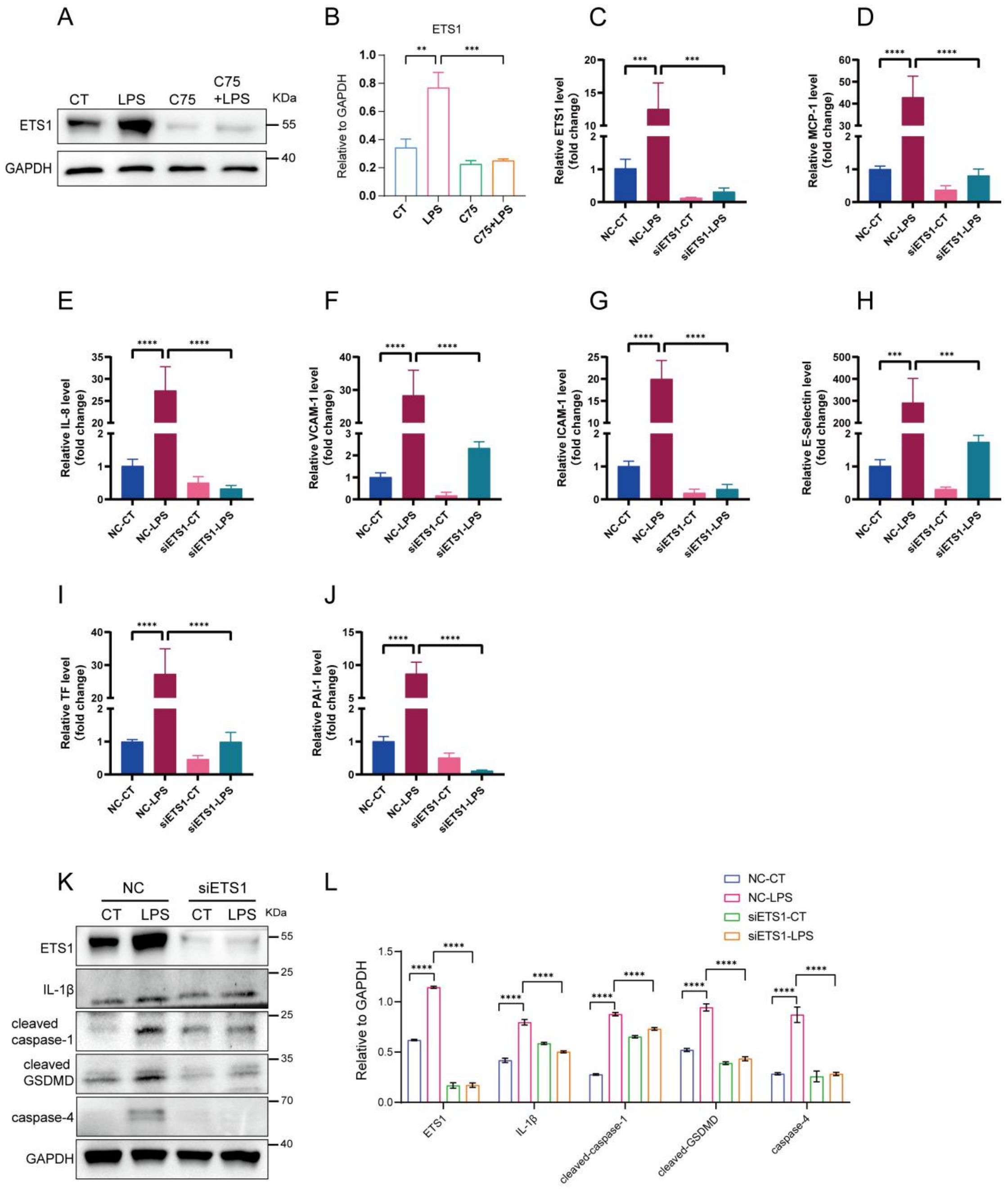

Supplementary Fig.3 The deletion of ETS1 inhibited EC activation induced by LPS

(A-B) The protein levels of ETS1 in LPS-stimulated HUVEC with or without C75 pretreatment were analyzed by western blot (n = 3). **(C-J)** HUVEC were transfected with siRNA specific to ETS1 for 48 h before LPS stimulation. The mRNA levels of ETS1, MCP-1, IL-8, VCAM-1, ICAM-1, E-selectin, TF and PAI-1 were detected by RT-qPCR (n = 3). **(K-L)** Representative immunoblots of cleaved IL-1β, cleaved caspase 1 and cleaved GSDMD with ETS1 knockdown before LPS stimuli in HUVEC (n = 3). All data were expressed as the mean ± SD. Comparison among three or more groups was analyzed by one-way ANOVA. ns, no significance, *p < 0.05, **p < 0.01, ***p < 0.001, and ****p < 0.0001.

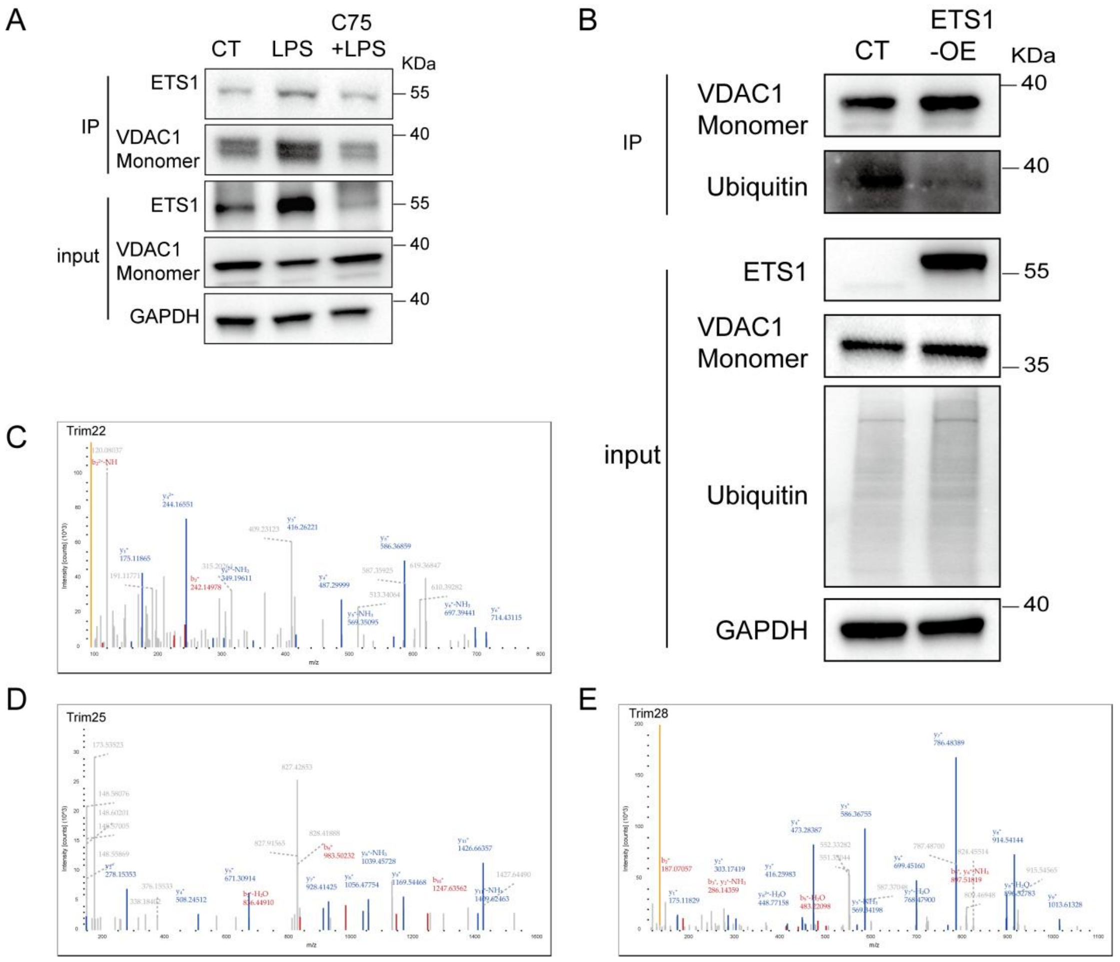

Supplementary Fig.4 The overexpression of ETS1 inhibited VDAC1 ubiquitination

(A) Immunoblot showing the binding of ETS1 with VDAC1. **(B)** HUVEC were transfected with adenovirus carrying human ETS1 construct or empty adenovirus for 48 h. The ubiquitination levels of VDAC1 were detected by western blot (n = 3). **(C-E)** MS image of Trim22, Trim25, Trim28.
